# Quadriceps tendon-bone vs all soft-tissue autografts for primary anterior cruciate ligament reconstruction: a systematic review and meta-analysis of 7,748 patients

**DOI:** 10.1530/EOR-2024-0204

**Published:** 2025-12-05

**Authors:** Konrad Malinowski, Dong Woon Kim, Jan Surma, Zygimantas Macius, Luke Tollefson, Robert F LaPrade, Przemysław Pękala, Marcin Mostowy

**Affiliations:** ^1^ International Evidence-Based Anatomy Working Group, Department of Anatomy, Jagiellonian University Medical College, Kraków, Poland; ^2^ Artromedical Orthopedic Clinic, Bełchatów, Poland; ^3^ Twin Cities Orthopedics, Edina, Minnesota, USA; ^4^ Faculty of Medicine and Health Sciences, Andrzej Frycz Modrzewski Kraków University, Kraków, Poland; ^5^ Lesser Poland Orthopedic and Rehabilitation Hospital, Kraków, Poland; ^6^ Orthopedic and Trauma Department, Veterans Memorial Teaching Hospital in Lodz, Medical University of Lodz, Lodz, Poland

**Keywords:** anterior cruciate ligament, quadriceps tendon graft, anterior cruciate ligament reconstruction, autograft, S-QT, B-QT

## Abstract

**Purpose:**

- The purpose of this systematic review and meta-analysis was to synthesise available evidence on functional outcomes, knee stability, and complications between quadriceps tendon with patellar bone block (B-QT) and quadriceps soft-tissue (S-QT) autografts for primary anterior cruciate ligament reconstructions (ACLR).

**Methods:**

- A systematic search (PROSPERO ID: CRD42023472220) was conducted according to the Preferred Reporting Items for Systematic Reviews and Meta-Analyses (PRISMA) guidelines and was performed on PubMed/MEDLINE, EMBASE, and WoS. The data concerning functional outcomes, knee stability, and complications were analysed.

**Results:**

- There were 5,898 B-QT patients (67.5% male) and 1,849 S-QT patients (51.5% male) included, with a mean age of 27.4 ± 9.3 and 24.7 ± 9.2 years and mean follow-up of 28.9 months (5.3–123.6 months) and 25.3 months (6.0–69.9 months), respectively. In a meta-analysis, the B-QT had higher KOOS subscales scores: activity-of-daily-living 95.98 (95% CI: 94.09–97.88) vs 93.88 (95% CI: 93.66–94.11), *P* = 0.028; pain 91.99 (95% CI: 89.48–94.49) vs 88.51 (95% CI: 88.21–88.81), *P* = 0.005; sports & recreation 84.26 (95% CI: 79.20–89.31) vs 79.21 (95% CI: 78.69–79.73), *P* = 0.041; symptoms 87.82 (95% CI: 84.75–90.90) vs 81.26 (95% CI: 80.71–81.82), *P* < 0.001; quality-of-life 78.28 (95% CI: 73.68–82.89) vs 65.37 (95% CI: 60.59–70.15), *P* < 0.001; and a higher Marx score: 11.90 (95% CI: 10.92–12.87) vs 9.65 (95% CI: 8.21–11.09), *P* = 0.006. B-QT had higher side-to-side difference in anteroposterior tibial translation: 1.55 mm (95% CI: 1.33–1.77) vs 1.15 mm (95% CI: 0.85–1.33 mm), *P* = 0.026.

**Conclusion:**

- A QT autograft, with or without a bone block, provides satisfactory functional outcomes, knee stability, and few complications in primary ACLR. B-QT may result in higher patient-reported outcomes, while S-QT may potentially result in slightly improved knee stability.

## Background

The quadriceps tendon autograft is becoming increasingly popular for both primary and revision anterior cruciate ligament reconstructions (ACLR) (1, 2). This may be due to several reasons, including its length, thickness versatility, variability in fixation methods, and a growing body of evidence demonstrating excellent patient outcomes (3). The quadriceps tendon graft can be harvested with a patellar bone block (B-QT) or as an all-soft tissue graft without a patellar bone block (S-QT) (4). While B-QT provides the benefit of improved graft bone-to-bone incorporation (5, 6), it is associated with a potential risk of a patellar fracture (7). The impact of using B-QT vs S-QT on the functional outcomes, knee stability, and risk of complications is not well known. Although a few systematic reviews on the topic of B-QT and S-QT autografts exist (8, 9), to the authors’ knowledge, no meta-analysis was published to date.

The purpose of this systematic review and meta-analysis was to synthesise available evidence on functional outcomes, knee stability, and complications between B-QT and S-QT autografts for primary ACLRs.

## Methods

The Preferred Reporting Items for Systematic Reviews and Meta-Analysis (PRISMA) guidelines were followed. This systematic review was registered on the PROSPERO International Prospective Register of Systematic Reviews (CRD42023472220).

### Searches

A systematic search was performed on PubMed/MEDLINE, EMBASE, and Web of Science databases on articles from their inception up to April 2024 by two independent reviewers (JS, ZM). The following search string was used (‘anterior cruciate ligament reconstruction’ OR ‘anterior cruciate ligament’ OR ACL) AND (quadriceps OR ‘quadriceps tendon’ OR QT) AND (autograft OR graft OR autologous). Two reviewers (JS, ZM) independently screened all retrieved articles by their titles and abstracts. A decision on which articles to include into the full-text evaluation stage was reached by consensus. Full-text evaluation of the articles was performed by the same two reviewers independently, and a decision on which articles to include was reached again by consensus. In case of any inconsistencies between the reviewers, the senior author (KM) was consulted for resolution. Further studies that might have been missed were manually searched by going through the reference lists of relevant articles.

### Study inclusion and exclusion criteria

Randomised controlled trials (RCTs), prospective and retrospective cohort studies (including non-comparative and comparative studies), case–control studies, and case series were included. All studies that were conducted on adult (skeletally mature) patients treated for primary ACLR using B-QT and S-QT autograft were included. Biomechanical studies, cadaveric studies, case reports, editorial articles, literature reviews, conference abstracts, and studies that did not report data on clinical and functional outcomes or complications were excluded. Revision ACLR or ACLR using other grafts were excluded. The studies were divided into two subgroups: B-QT vs S-QT, and patient-reported outcome measures, objective outcomes, and complications were recorded.

### Study quality assessment

For observational studies, the quality was evaluated using the methodological index for non-randomised studies (MINORS) score (10). The MINORS score is a summation of individual item scores (from zero to two for each item), with a maximum of 16 for non-comparative studies and 24 for comparative studies. According to the MINORS scale, studies with an overall score of 11 or more (non-comparative) or 17 or more (comparative) are rated as ‘high quality’, while those with a score of less than 11 (non-comparative) or less than 17 (comparative) are rated as ‘low quality’. The revised Cochrane Risk of Bias tool (RoB 2.0) was used for randomised controlled trials (11). RoB 2.0 addresses five specific domains: i) bias arising from the randomisation process, ii) bias due to deviations from intended interventions, iii) bias due to missing outcome data, iv) bias in measurement of the outcome, and v) bias in selection of the reported result. Two reviewers (JS, ZM) independently applied the tool to each included study and, following the algorithms provided in the guidance for RoB 2.0 usage, derived overall risk of bias results for each specific domain, as well as for each study (Fig. 2).

### Data extraction strategy

The data were extracted from the selected articles by two reviewers (JS, ZM) and checked by another (initials blinded for review) using a standardised data extraction form created with Microsoft Access^®^ (Version 2021, Microsoft Corp, Redmond Washington). Patient-reported outcome measures (Lysholm score, subjective International Knee Documentation Committee [IKDC] score, knee injury and osteoarthritis outcome score (KOOS) subscale scores, Marx score, and Tegner activity level) were extracted, as were clinical outcomes (objective IKDC knee evaluation, pivot-shift test, Lachman test, and KT-1000/2000/Rolimeter arthrometer side-to-side difference (SSD) in anteroposterior laxity). Isometric and isokinetic (at 60°/s, 90°/s, 180°/s, and 240°/s) peak extensor and flexor torque, and the single leg triple hop test, expressed as limb symmetry indexes (LSIs), were recorded. In terms of complications, the visual analogue scale (VAS) score for pain, and the incidences of donor site morbidity, arthrofibrosis, patellar fractures, and graft ruptures were extracted.

For each study, the graft type, study design, level of evidence, study sample size, and participant characteristics such as age, gender, and duration of follow-up were recorded. In studies in which grafts other than quadriceps tendons were used for ACL reconstruction, only patients treated with quadriceps tendon autograft were considered. Studies with a minimum follow-up of 6 months were eligible. Where studies reported outcomes for multiple follow-up periods, data from the final study follow-up were extracted. In cases where a single outcome was reported from multiple publications arising from the same study population, the study with the greatest number of patients was included into the review.

### Data synthesis and presentation

All statistical analyses were performed using R^®^ Statistical Software (Version 4.3.1, R Foundation for Statistical Computing, Austria). For continuous outcomes, the mean ± standard deviation (SD) was extracted to be used for a random-effects meta-analysis. Where not reported, the estimated sample mean ± SD was calculated from available data based on previously published and validated protocols by Luo *et al.* (12) and Wan *et al.* (13), respectively. As we anticipated considerable between-study heterogeneity due to varying study-level factors such as patient age, sex, and length of follow-up, the random-effects model was used to pool the means. The restricted maximum likelihood estimator (14) was used to calculate the heterogeneity variance *τ*^2^, and Knapp–Hartung adjustments (15) to calculate confidence intervals around the pooled means. For dichotomous outcomes, the proportions were logit-transformed and pooled using the generalised linear mixed-effects model (16). The maximum likelihood estimator (14) was used to calculate the heterogeneity variance *τ*^2^, and the Knapp–Hartung adjustments (15) to calculate confidence intervals. Multivariate regression analysis was performed to investigate the effects of follow-up length on the outcomes between B-QT and S-QT.

## Results

### Study inclusion

**Figure 1:**
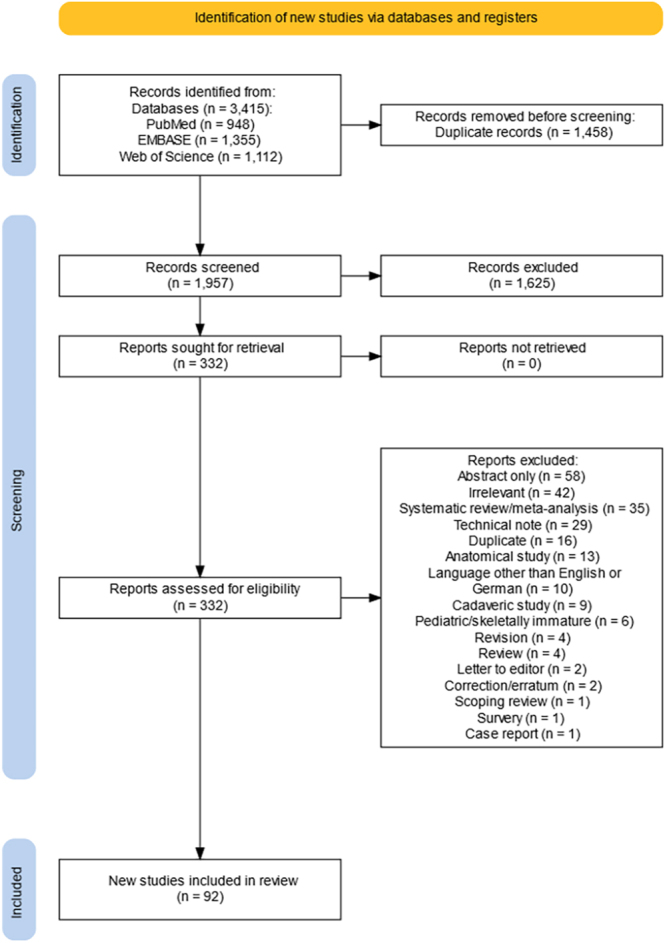
PRISMA flow diagram of study inclusion and exclusion.

Preliminary search identified a total of 3,415 records across all databases. After removing 1,458 duplicates, the titles and abstracts of the remaining 1,957 records were screened. Afterwards, 332 full texts were screened for eligibility, and finally 92 records were included in the review (Fig. 1).

### Characteristics of included studies

Thirteen studies were randomised controlled trials (17, 18, 19, 20, 21, 22, 23, 24, 25, 26, 27, 28), ten were prospective comparative studies (29, 30, 31, 32, 33, 34, 35, 36, 37, 38), six were prospective cohort studies (17, 39, 40, 41, 42, 43), 39 were retrospective comparative studies (21, 31, 43, 44, 45, 46, 47, 48, 49, 50, 51, 52, 53, 54, 55, 56, 57, 58, 59, 60, 61, 62, 63, 64, 65, 66, 67, 68, 69, 70, 71, 72, 73, 74, 75, 76), nine were retrospective cohort studies (17, 42, 43, 77, 78, 79, 80, 81, 82), four were case–control studies (83, 84, 85, 86), and 11 were case series (87, 88, 89, 90, 91, 92, 93, 94, 95, 96). Sixty-two studies utilised quadriceps tendon autografts with bone block (B-QT), while 30 studies utilised all soft-tissue quadriceps tendon autografts without a bone block (S-QT). A total of 5,898 B-QT patients (3,980 male, 1,593 female) and 1,849 S-QT patients (953 male, 642 female) were included in this study. The mean age was 27.4 ± 9.3 and 24.7 ± 9.2 years in the B-QT and S-QT groups, respectively. The mean time to follow-up was 28.9 months (range: 5.3–123.6 months) and 25.3 months (range: 6.0–69.9 months) in the B-QT and S-QT groups, respectively (Tables 1 and 2).

**Table 1:** Characteristics of included B-QT studies.

Study	Country	Study design	LOE	Subjects, *n*			Age	Follow-up	MINORS
				Total	Male	Female			
Akoto & Hoeher (39)	Germany	PCS	II	87	75	12	31.0 ± 7.6	12+	10/16
Akoto *et al.* (29)	Germany	PCOS	II	10	10	0	27.4 ± 10.7	12.7 ± 3.9	19/24
Akoto *et al.* (44)	Germany	RCOS	III	41	32	9	29.0 ± 10.0	14.0 ± 1.4	17/24
Barié *et al.* (99)	Germany	PCS	II	106	63	43	30.0 ± 5.4	12.4 ± 0.4	11/16
Barié *et al.* (100)	Germany	RCS	III	69	42	27	27.0 ± 6.1	90.0 ± 4.5	12/16
Barié *et al.* (17)	Germany	RCT	I	30	17	13	30.5 ± 7.8	123.6 ± 3.0	
Cavaignac *et al.* (47)	Switzerland	RCOS	III	45	25	20	32.1 ± 8.0	40.8 ± 7.2	16/24
Chen *et al.* (87)	Taiwan	CS	IV	34	22	21	26.0 ± 9.3	62.0 ± 8.6	12/16
Csapo *et al.* (77)	Austria	RCS	III	46	20	26	20.7 ± 3.0	5.3 ± 0.8	14/16
Fischer *et al.* (48)	Austria	RCOS	III	61	34	27	21.6 ± 7.4	7.6 ± 1.4	16/24
Fu *et al.* (89)	USA	CS	IV	57	38	19	21.7 ± 7.3	24+	9/16
Galan *et al.* (78)	Argentina	RCS	III	291	268	23	23.2 ± 4.4	60+	9/16
Geib *et al.* (90)	USA	CS	IV	157	80	77	31.7	59+	8/16
Gorschewsky *et al.* (49)	Switzerland	RCOS	III	193	122	71	33.0 ± 17.2	29.0 ± 7.2	18/24
Gorschewsky *et al.* (49)	Switzerland	RCOS	III	93	65	28	32.0 ± 8.0	34+	18/24
Goto *et al.* (91)	Switzerland	CS	IV	73	0	73	33.8 ± 11.5	9.0 ± 2.3	18/24
Guimarães *et al.* (41)	Brazil	PCS	II	17	15	2	28.5 ± 6.6	120+	11/16
Guney-Deniz *et al.* (83)	Turkey	CCS	III	22	17	5	27.8 ± 2.8	13.5 ± 2.1	20/24
Han & Lee (51)	South Korea	RCOS	III	72	68	4	27.8 ± 7.6	39.7 ± 13.9	20/24
Herman *et al.* (52)	USA	RCOS	III	14	0	14	19.7 ± 5.4	54.0 ± 12.0	18/24
Hofer *et al.* (79)	USA	RCS	III	31	12	19	19.0 ± 3.4	12+	10/16
Horstmann *et al.* (18)	Germany	RCT	I	24	21	3	24.1 ± 3.6	24+	
Horteur *et al.* (92)	France	CS	IV	25	20	5	31.6 ± 11.0	7.3 ± 2.0	17/24
Irrgang *et al.* (19)	USA	RCT	I	57	38	19	21.7 ± 7.3	24+	
Jacquet *et al.* (55)	France	RCOS	III	91	65	26	29.9 ± 8.0	37+	18/24
Kaarre *et al.* (101)	USA	RCOS	III	79	44	35	22.8 ± 7.5	9.0 ± 1.6	16/24
Karpinski *et al.* (30)	Germany	PCOS	II	25	16	9	31.7 ± 15.2	24+	22/24
Kim *et al.* (59)	South Korea	RCOS	III	29	11	18	25.3 ± 5.1	24+	17/24
Kim *et al.* (60)	South Korea	RCOS	III	59	46	13	25.9 ± 8.2	24+	17/24
Kim *et al.* (31)	South Korea	PCOS	II	142	117	25	29.8 ± 10.7	24+	18/24
Kim *et al.* (58)	South Korea	RCOS	III	75	63	12	28.4 ± 10.5	17.9 ± 6.1	17/24
Komzák *et al.* (20)	Czech Republic	RCT	I	40	21	19	29.8 ± 7.4	28.0 ± 2.1	
Kuangyang *et al.* (61)	China	RCOS	III	33	27	6	28.8 ± 6.5	12+	16/24
Kwak *et al.* (85)	South Korea	CCS	III	45	38	7	34.5 ± 12.8	29.8 ± 6.5	16/24
Lee *et al.* (94)	South Korea	CS	IV	67	58	9	28.0 ± 8.0	41.0 ± 5.0	8/16
Lee *et al.* (43)	South Korea	RCS	III	137	123	14	27.0 ± 6.7	59.0 ± 6.9	8/16
Lee *et al.* (43)	South Korea	PCS	II	247	219	28	29.0 ± 7.5	44.0 ± 11.1	10/16
Lee *et al.* (102)	South Korea	RCOS	III	104	89	15	29.3 ± 8.2	32.3 ± 8.0	17/24
Lee *et al.* (62)	South Korea	RCOS	III	48	44	4	31.1 ± 9.0	35.6 ± 8.3	16/24
Lee *et al.* (93)	South Korea	CS	IV	139	119	20	30.0 ± 6.5	122.4 ± 31.2	20/24
Lind *et al.* (32)	Denmark	PCOS	III	531	372	159	26.2	12+	18/24
Lind *et al.* (32)	Denmark	RCT	I	50	29	21	27.2 ± 6.4	24+	
Lind *et al.* (21)	Denmark	RCOS	III	1,194	776	418	25.5 ± 8.3	12+	20/24
Lubis & Dastril (33)	Indonesia	PCOS	III	15	15	0	28.0 ± 6.3	12+	17/24
Lund *et al.* (22)	Denmark	RCT	I	21	17	4	31.0 ± 8.0	24+	
Malinowski *et al.* (65)	Poland	RCT	III	106	NR		NR	18+	17/24
Mouarbes *et al.* (66)	France	RCOS	III	30	28	2	23.9 ± 7.1	12+	16/24
Mutsuzaki *et al.* (80)	Japan	RCS	III	8	2	6	41.6 ± 10.6	28.8 ± 18.0	7/16
Ollivier *et al.* (95)	France	CS	IV	340	278	62	26.0 ± 8.0	24.0 ± 8.4	9/16
Ortmaier *et al.* (81)	Austria	RCS	III	20	NR		30.9 ± 13.2	21.0 ± 3.8	12/16
Pigozzi *et al.* (103)	Italy	RCT	I	24	17	7	33.0 ± 6.7	6+	
Pomenta *et al.* (35)	Spain	PCOS	II	20	17	3	30.2 ± 2.4	27.4 ± 3.2	16/24
Runer *et al.* (37)	Austria	PCOS	III	40	23	17	34.6 ± 11.0	24+	14/24
Runer *et al.* (69)	Austria	RCOS	III	217	127	90	28.9 ± 11.9	24+	19/24
Runer *et al.* (36)	Austria	PCS	II	45	29	16	28.9 ± 11.6	78.9 ± 13.6	18/24
Schwery *et al.* (72)	USA	RCOS	III	17	NR		18.5 ± 5.1	9.2 ± 0.6	16/24
Setliff *et al.* (73)	USA	RCOS	III	48	37	11	21.7 ± 6.3	17.0 ± 7.9	18/24
Sinding *et al.* (24)	Denmark	RCT	I	42	25	17	28.7 ± 6.4	12.4 ± 0.6	
Sofu (74)	Turkey	RCOS	III	23	21	2	26.8	37.6 ± 16.1	16/24
Tashman *et al.* (26)	USA	RCT	I	57	38	19	21.7 ± 7.3	24+	
Vilchez-Cavazos *et al.* (28)	Mexico	RCT	I	14	11	3	24.5 ± 9.1	12+	
Yamasaki *et al.* (86)	Japan	CCS	III	21	12	9	20.4 ± 5.6	38.6 ± 24.8	18/24

LOE, level of evidence; MINORS, methodological index for non-randomised studies; RCT, randomised controlled trial; RCOS, retrospective comparative study; PCOS, prospective comparative study; RCS, retrospective cohort study; CS, case series; CCS, case–control study; PCS, prospective cohort study.

**Table 2:** Characteristics of included S-QT studies.

Study	Country	Study design	LOE	Subjects, *n*			Age	Follow-up	MINORS
				Total	Male	Female			
Aslam *et al.* (45)	India	RCOS	III	35	19	11	24.6 ± 3.3	11.3 ± 0.4	18/24
Brinkman *et al.* (46)	USA	RCOS	III	37	17	20	23.4 ± 7.0	69.9 ± 6.2	20/24
DeAngelis & Fulkerson (88)	USA	CS	IV	191	NR		NR	66.0 ± 14.9	10/16
Geib *et al.* (90)	USA	CS	IV	41	22	19	31.7	41+	8/16
Gille *et al.* (40)	Germany	PCS	II	54	45	9	31.0 ± 8.8	16.0 ± 2.6	11/16
Greif *et al.* (50)	USA	RCOS	III	124	96	28	29.5 ± 9.0	31.7 ± 5.9	18/24
Hogan *et al.* (53)	USA	RCOS	III	39	23	16	18.7 ± 6.3	22.4 ± 10.6	20/24
Hughes *et al.* (54)	USA	RCOS	III	29	15	14	23.0 ± 7.4	10.7 ± 2.0	17/24
Hunnicutt *et al.* (42)	USA	RCS	III	15	12	3	25.0 ± 7.8	10.2 ± 4.9	12/16
Hunnicutt *et al.* (104)	USA	PCS	II	320	156	164	18.1 ± 3.1	6+	13/16
Iriuchishima *et al.* (84)	Japan	CCS	III	20	2	18	49.0 ± 8.0	12+	8/16
Johnston *et al.* (57)	Australia	RCOS	III	37	29	8	21.0 ± 4.5	6+	17/24
Johnston *et al.* (105)	Australia	RCOS	III	35	28	7	20.0 ± 6.2	12+	17/24
Letter *et al.* (63)	USA	RCOS	III	17	11	6	25.8 ± 4.9	24.9 ± 13.5	18/24
Letter *et al.* (64)	USA	RCOS	III	26	21	5	27.4 ± 5.6	23.5 ± 11.0	18/24
Martin-Alguacil *et al.* (23)	Spain	RCT	I	26	23	3	18.7 ± 3.6	24+	
Panos *et al.* (34)	Australia	PCOS	II	23	16	7	22.0 ± 6.1	12+	17/24
Perez *et al.* (67)	USA	RCOS	III	28	17	11	23.1 ± 5.5	31.6 ± 4.7	16/24
Pichler *et al.* (82)	Austria	RCS	III	40	29	11	31.3 ± 9.5	16.8 ± 4.2	10/16
Renfree *et al.* (68)	USA	RCOS	III	32	13	19	17.6 ± 2.8	24+	18/24
Schagemann *et al.* (70)	Germany	RCOS	III	8	4	4	23.0 ± 13.0	69+	16/24
Schmücker *et al.* (71)	Denmark	RCOS	III	223	140	83	26.6 ± 8.6	27.2 ± 9.8	17/24
Schulz *et al.* (96)	Germany	CS	IV	55	31	24	31.7 ± 9.4	29.5 ± 3.1	12/16
Setliff *et al.* (73)	USA	RCOS	III	147	77	70	21.8 ± 7.4	17.0 ± 7.9	18/24
Tang *et al.* (25)	Turkey	RCT	I	17	17	0	28.1 ± 6.2	24+	
Tirupathi *et al.* (27)	India	RCT	I	44	NR		NR	24+	
Todor *et al.* (75)	Roumania	RCOS	III	39	26	13	30.6 ± 8.7	33.8 ± 6.6	14/24
von Essen *et al.* (38)	Sweden	PCOS	II	40	24	16	29.4 ± 7.9	6+	18/24
Zhou *et al.* (76)	New Zealand	RCOS	III	107	48	59	29.7 ± 11.9	24+	17/24

LOE, level of evidence; MINORS, methodological index for non-randomised studies; RCOS, retrospective comparative study; PCOS, prospective comparative study; RCS, retrospective cohort study; PCS, prospective cohort study; RCT, randomised controlled trial; CS, case series; CCS, case-control study.

### Methodological quality

**Figure 2:**
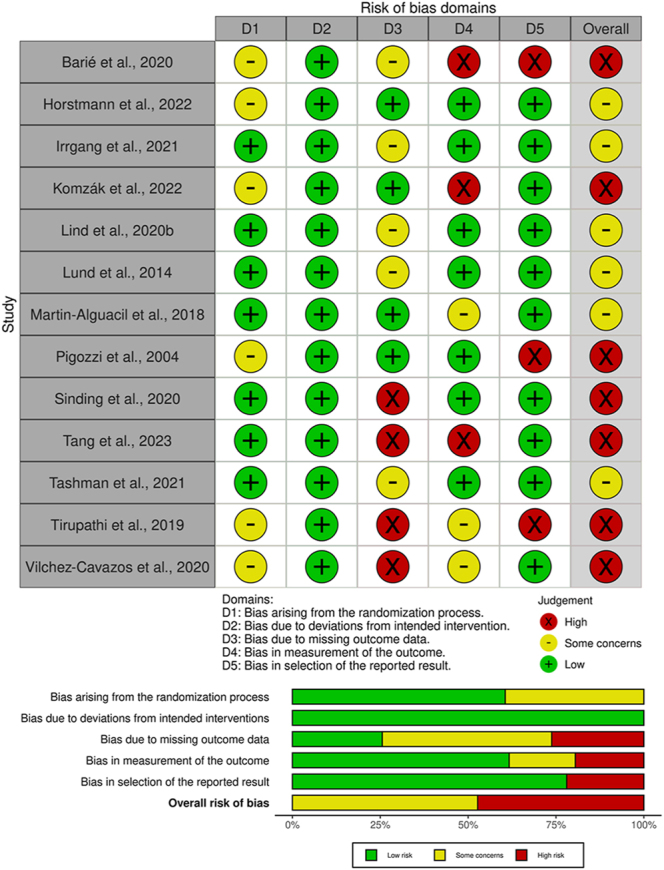
Results of Cochrane risk of bias (RoB 2.0) assessment tool for randomised controlled trials studies.

Of the 13 RCTs, the risk of bias was assessed as ‘high risk’ in seven, ‘some concerns’ in six, and ‘low risk’ in none (Fig. 2). Of the 79 non-randomised studies, the median MINORS score was 10 out of 16 (range: 7–14) for the 55 non-randomised cohort studies, and 17 out of 24 (range: 14–22) for the 24 non-randomised comparative studies (Tables 1 and 2).

### Subjective outcomes – univariate meta-analysis

**Figure 3:**
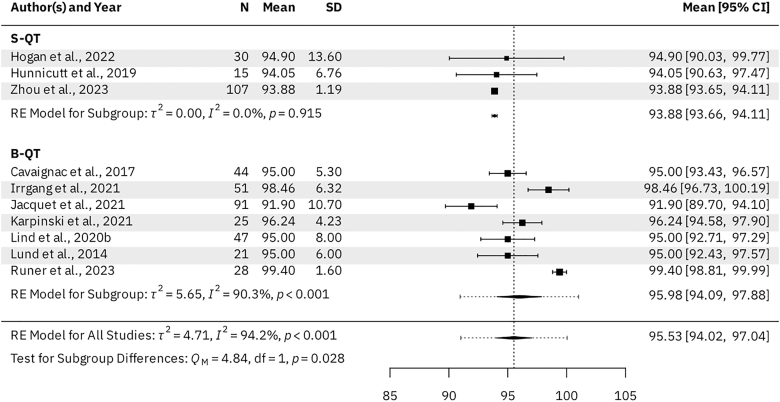
Forest plot of KOOS activities of daily living (ADL) subscale scores.

**Figure 4:**
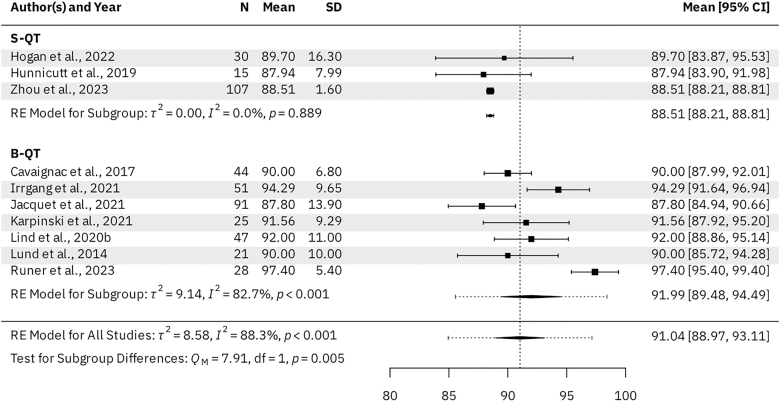
Forest plot of KOOS pain subscale scores.

**Figure 5:**
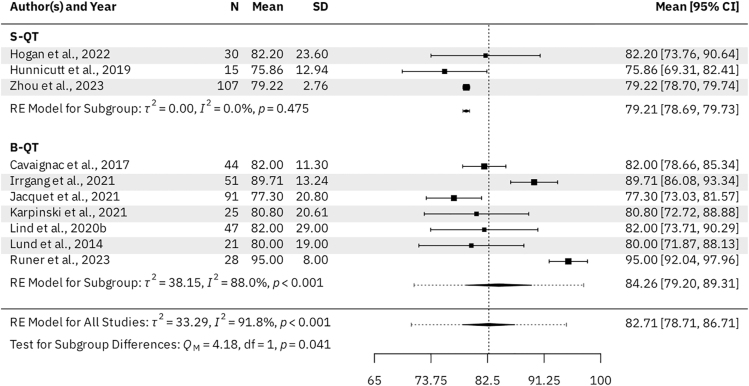
Forest plot of KOOS sports & recreation subscale scores.

**Figure 6:**
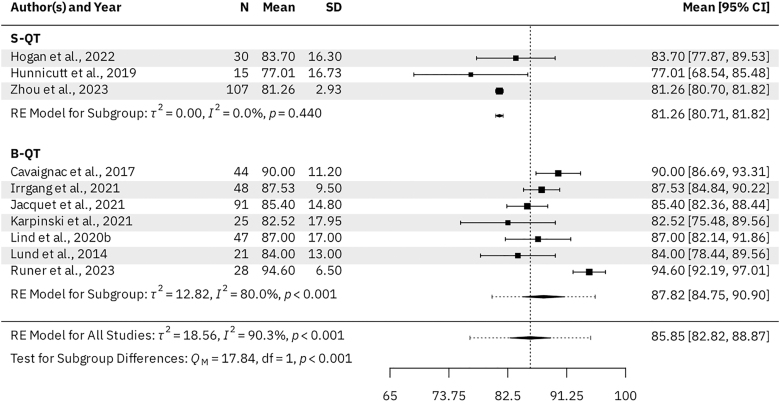
Forest plot of KOOS symptoms subscale scores.

**Figure 7:**
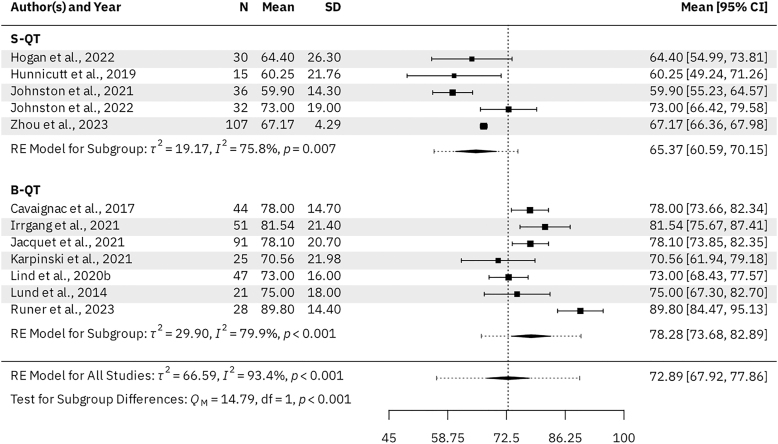
Forest plot of KOOS quality-of-life subscale scores.

**Figure 8:**
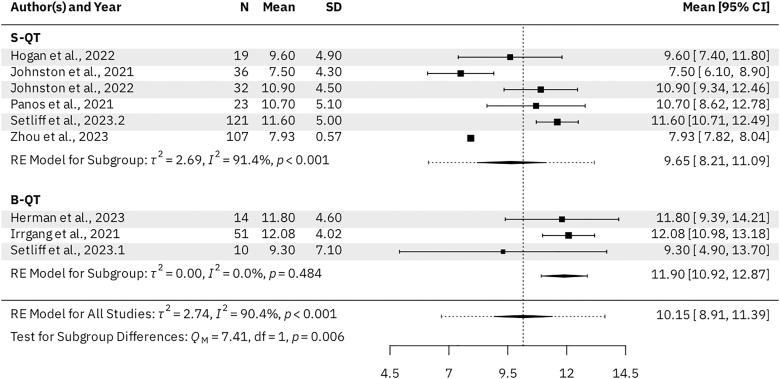
Forest plot of Marx activity scores.

Seven B-QT studies (*n* = 334 patients) and three S-QT studies (*n* = 161 patients) reported KOOS subscale scores for the activities of daily living (ADL), pain, sports & recreation, and symptoms subscales. For the B-QT and S-QT groups, the results were as follows. The mean KOOS ADL was 95.98 (95% CI: 94.09–97.88) vs 93.88 (95% CI: 93.66–94.11, *P* = 0.028), respectively (Fig. 3). The mean KOOS Pain was 91.99 (95% CI: 89.48–94.49) vs 88.51 (95% CI: 88.21–88.81, *P* = 0.005), respectively (Fig. 4). The mean KOOS Sports & Recreation was 84.26 (95% CI: 79.20–89.31) vs 79.21 (95% CI: 78.69–79.73, *P* = 0.041), respectively (Fig. 5). The mean KOOS Symptoms was 87.82 (95% CI: 84.75–90.90) vs 81.26 (95% CI: 80.71–81.82, *P* < 0.001), respectively (Fig. 6). Seven B-QT studies (*n* = 334 patients) and five S-QT studies (*n* = 233 patients) reported KOOS quality-of-life subscale scores. The pooled mean KOOS quality-of-life was 78.28 (95% CI: 73.68–82.89) vs 65.37 (95% CI: 60.59–70.15, *P* < 0.001), respectively (Fig. 7). Three B-QT studies (*n* = 119 patients) and six S-QT studies (*n* = 388 patients) reported Marx activity scores at a mean follow-up of 24.7 months (range: 17.0–54.0 months) and 17.7 months (range: 6.0–24.0 months), respectively. The B-QT group had a significantly higher Marx score than the S-QT group, with pooled mean Marx scores of 11.90 (95% CI: 10.92–12.87) vs 9.65 (95% CI: 8.21–11.09, *P* = 0.006), respectively (Fig. 8).

The rest of the subjective outcomes did not differ significantly between B-QT and S-QT. Twenty-seven B-QT studies (*n* = 1,420 patients) and 16 S-QT studies (*n* = 894 patients) reported subjective IKDC scores at a mean follow-up of 39.4 months (range: 12.0–123.6 months) and 33.1 months (range: 6.0–69.9 months), respectively. The mean subjective IKDC scores were 84.45 (95% CI: 81.97–86.92) and 83.97 (95% CI: 80.23–87.72) for the B-QT and S-QT groups, respectively (*P* = 0.837, Supplementary Fig. 1 (see section on Supplementary materials given at the end of the article)).

Thirty-two B-QT studies (*n* = 2,295 patients) and 14 S-QT studies (*n* = 527 patients) reported Lysholm scores at a mean follow-up of 43.6 months (range: 12.0–123.6 months) and 28.8 months (range: 10.2–69.9 months) respectively. The pooled mean Lysholm scores were 91.45 (95% CI: 90.51–92.40) and 89.41 (95%–CI: 87.25–91.58) for the B-QT and S-QT groups, respectively (*P* = 0.095, Supplementary Fig. 2).

Nineteen B-QT studies (*n* = 1,397 patients) and nine S-QT studies (*n* = 398 patients) reported Tegner scores at a mean follow-up of 49.0 months (range: 12.4–123.6 months) and 25.2 months (10.2–31.7 months), respectively. The pooled mean Tegner scores were 5.96 (95% CI: 5.41–6.51) and 5.40 (95% CI: 4.62–6.18) for the B-QT and S-QT groups, respectively (*P* = 0.238, Supplementary Fig. 3).

### Objective outcomes – univariate meta-analysis

**Figure 9:**
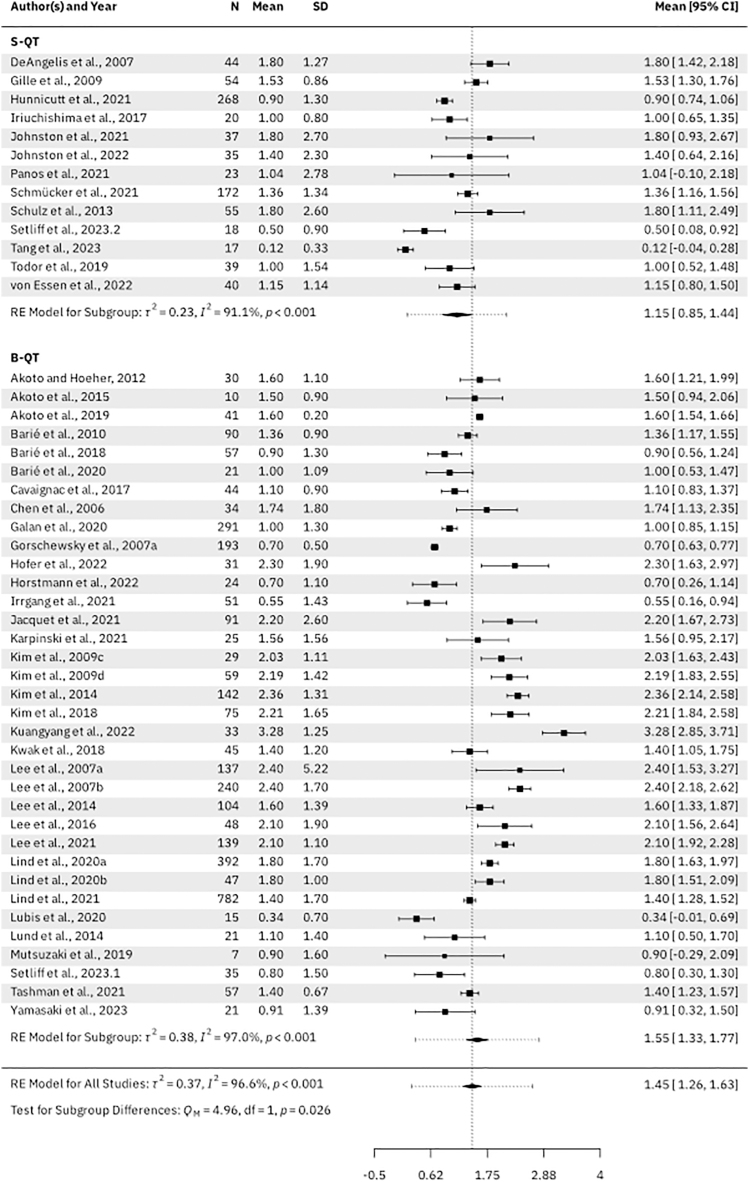
Forest plot of instrumental anterior knee laxity.

Thirty-five B-QT studies (*n* = 4,137 patients) and 13 S-QT studies (*n* = 1,201 patients) reported instrumental anterior knee laxity using an arthrometer (e.g. KT-1000, KT-2000 or a Rolimeter) at a mean follow-up of 29.4 months (range: 12.0–123.6 months) and 23.9 months (range: 6.0–66.0 months), respectively. The B-QT had a significantly increased side-to-side difference in anteroposterior tibial translation: 1.55 mm (95% CI: 1.33–1.77 mm) vs 1.15 mm (95% CI: 0.85–1.33 mm), *P* = 0.026 (Fig. 9).

The rest of the objective outcomes did not differ significantly between B-QT and S-QT. Nineteen B-QT studies (*n* = 1,860 patients) and four S-QT studies (*n* = 170 patients) reported objective IKDC assessments at a mean follow-up of 39.9 months (range: 12.0–90.0 months) and 25.8 months (range: 12.0–40.5 months), respectively. The pooled prevalence of objective IKDC grade C (abnormal) or D (severely abnormal) was 9.3% (95% CI: 6.8–12.6%) and 9.7% (95% CI: 2.8–28.9%) for the B-QT and S-QT groups, respectively (*P* = 0.945, Supplementary Fig. 4).

Twenty-eight B-QT studies (*n* = 2,148 patients) and six S-QT studies (*n* = 807 patients) reported Lachman assessments at a mean follow-up of 44.0 months (range: 12.0–122.4 months) and 24.5 months (range, 6.0–66.0 months) for the B-QT and S-QT groups, respectively. The pooled prevalence of Lachman grade ≥1+ (i.e. ≥3 mm) was 19.5% (95% CI: 15.4–24.3%) and 13.0% (95% CI: 5.3–28.4%) in the B-QT and S-QT groups, respectively (*P* = 0.428, Supplementary Fig. 5).

Thirty-one B-QT studies (*n* = 3,512 patients) and seven S-QT studies (*n* = 580 patients) reported pivot-shift assessments at a mean follow-up of 27.9 months (range: 12.0–122.4 months) and 22.7 months (range: 6.0–40.5 months) for the B-QT and S-QT groups, respectively. The pooled prevalence of pivot-shift grade ≥1+ (i.e. glide) was 14.7% (95% CI: 10.3–20.6%) and 0.3% (95% CI: 0.00–19.3%) in the B-QT and S-QT groups, respectively (*P* = 0.071, Supplementary Fig. 6).

### Muscle strength recovery – univariate meta-analysis

No significant differences in muscle strength recovery were found between B-QT and S-QT (Supplementary Figs 7, 8, 9, 10, 11, 12, 13). Five B-QT studies (*n* = 309 patients) and four S-QT studies (*n* = 81 patients) reported isometric knee extensor muscle strength at a mean follow-up of 37.5 months (range: 9.0–62.0 months) and 13.9 months (range: 10.2–24.9 months) for the B-QT and S-QT groups, respectively. The pooled mean LSIs were 82.36% (95% CI: 75.45–89.28%) and 82.49% (95% CI: 75.61–89.38%) for the B-QT and S-QT groups, respectively (*P* = 0.980, Supplementary Fig. 7).

Eleven B-QT studies (*n* = 532 patients) and four S-QT studies (*n* = 407 patients) reported isokinetic knee extensor muscle strength (60°/s) at a mean follow-up of 21.4 months (range: 5.3–41.0 months) and 6.7 months (range: 6.0–12.0 months) for the B-QT and S-QT groups, respectively. The mean LSIs were 76.71% (95% CI: 73.15–80.27%) and 73.92% (95% CI: 65.73–82.12%) for the B-QT and S-QT groups, respectively (*P* = 0.522, Supplementary Fig. 8).

Eight B-QT studies (*n* = 352 patients) and three S-QT studies (87 patients) reported isokinetic knee extensor strength (180°/s) at a mean follow-up of 28.5 months (range: 7.3–41.0 months) and 9.1 months (range: 6.0–12.0 months) for the B-QT and S-QT groups, respectively. The mean (95% CI) LSIs were 83.89% (80.38–87.41%) and 80.86 (71.67–90.04%) for the B-QT and S-QT groups, respectively (*P* = 0.538, Supplementary Fig. 9).

Three B-QT studies (*n* = 155 patients) and two S-QT studies (*n* = 49 patients) reported isometric knee flexor strength at a mean follow-up of 21.5 months (range: 9.0–62.0 months) and 11.2 months (range: 10.7–12.0 months) for the B-QT and S-QT groups, respectively. The pooled mean LSIs were 94.40% (95% CI: 89.51–99.30%) and 97.96% (95% CI: 93.30–102.62%) for the B-QT and S-QT groups, respectively (*P* = 0.213, Supplementary Fig. 10).

Seven B-QT studies (*n* = 317 patients) and three S-QT studies (*n* = 392 patients) reported isokinetic knee flexor muscle strength (60°/s) at a mean follow-up of 12.9 months (range: 5.3–35.6 months) and 6.5 months (range: 6.0–12.0 months) for the B-QT and S-QT groups, respectively. The pooled mean LSIs were 94.43% (95% CI: 92.43–96.43%) and 94.94% (95% CI: 90.95–98.93%) for the B-QT and S-QT groups, respectively (*P* = 0.797, Supplementary Fig. 11).

Four B-QT studies (*n* = 137 patients) and two S-QT studies (72 patients) reported isokinetic knee flexor strength (180°/s) at a mean follow-up of 19.8 months (range: 7.3–35.6 months) and 8.9 months (range: 6.0–12.0 months) for the B-QT and S-QT groups, respectively. The mean LSIs were 97.75% (95% CI: 94.95–100.56%) and 99.47% (95% CI: 95.08–103.87%) for the B-QT and S-QT groups, respectively (*P* = 0.291, Supplementary Fig. 12).

Seven B-QT studies (*n* = 318 patients) and six S-QT studies (*n* = 387 patients) reported single-leg triple hop tests at a mean follow-up of 23.8 months (range: 12.0–78.9 months) and 41.1 months (range: 6.0–66.0 months) for the B-QT and S-QT groups, respectively. The pooled mean LSIs were 93.23% (95% CI: 91.30–95.17%) and 91.68% (95% CI: 87.51–95.84%) for the B-QT and S-QT groups, respectively (*P* = 0.500, Supplementary Fig. 13).

### Complications – univariate meta-analysis

No significant differences in complications were found between B-QT and S-QT (Supplementary Figs 14, 15, 16, 17, 18). Thirty-seven B-QT studies (*n* = 3,556 patients) and 19 S-QT studies (*n* = 1,622 patients) reported graft ruptures at a mean (range) follow-up of 36.8 (12.0–123.6) and 26.6 (6.0–69.9) months for the B-QT and S-QT groups, respectively. The prevalence (95% CI) of graft ruptures was 2.5% (1.6–3.7%) and 1.9 (1.0–3.4%) for the B-QT and S-QT groups, respectively (*P* = 0.458, Supplementary Fig. 14).

Sixteen B-QT studies (*n* = 1,231 patients) and ten S-QT studies (*n* = 936 patients) reported arthrofibrosis at a mean (range) follow-up of 44.6 (12.4–123.6) and 20.8 (6.0–69.9) months for the B-QT and S-QT groups, respectively. The prevalence (95% CI) of arthrofibrosis was 3.6% (2.0–6.2%) and 5.8 (4.3–7.5%) for the B-QT and S-QT groups, respectively (*P* = 0.126, Supplementary Fig. 15).

Thirteen B-QT studies (*n* = 1,217 patients) and three S-QT studies (*n* = 214 patients) reported the prevalence of patellar fractures at a mean (range) follow-up of 49.0 (17.0–123.6) and 19.9 (17.0–31.6) months for the B-QT and S-QT groups, respectively. The prevalence (95% CI) of patellar fracture was 2.0% (1.0–4.3%) for the B-QT group, and no cases were found in the three S-QT studies that reported the prevalence of patellar fractures in their cohorts (*P* = 1.000, Supplementary Fig. 16).

Twenty-nine B-QT studies (*n* = 2,267 patients) and ten S-QT studies (*n* = 628 patients) reported donor-site morbidity at a mean (range) follow-up of 41.1 (9.0–123.6) and 35.9 (6.0–66.0) months for the B-QT and S-QT groups, respectively. The prevalence (95% CI) of donor-site morbidity was 6.6% (4.4–10.0%) and 3.7% (0.4–24.7%) for the B-QT and S-QT groups, respectively (*P* = 0.580, Supplementary Fig. 17).

Seven B-QT studies (*n* = 424 patients) and five S-QT studies (*n* = 267 patients) reported VAS scores for pain at a mean (range) follow-up of 28.9 (12.0–78.9) and 16.3 (6.0–68.6) months for the B-QT and S-QT groups, respectively. The mean (95% CI) VAS scores were 0.93 (0.53–1.31) and 1.40 (0.39–2.40) for the B-QT and S-QT groups, respectively (*P* = 0.401, Supplementary Fig. 18).

### Multivariate regression analysis

The multivariate regression revealed only one variable in which the follow-up length significantly influenced the results: extensor muscle-strength limb symmetry index in isokinetic 60°/s (*β* = 2.23, *P* = 0.03). All results of the multivariate regression were summarised in the Table 3.

**Table 3:** Multivariate regression assessing the impact of follow-up length on the differences between B-QT and S-QT subgroups in given variables. Bold *P* value indicates statistical significance at *P < *0.05.

	*β*	*P*
KOOS		
ADL	−0.05	0.84
Pain	−0.03	0.91
Sport	0.11	0.83
Symptoms	0.14	0.68
Quality-of-life	0.00	0.99
Marx	−0.03	0.83
IKDC subjective	0.07	0.52
Lysholm	0.04	0.40
Tegner	0.51	0.39
Instrumental laxity (SSD, mm)	0.01	0.39
IKDC objective (grade C/D)	0.06	0.13
Lachman (grade ≥1+)	0.01	0.44
Pivot shift (grade ≥1+)	0.02	0.72
Extensor muscle strength (LSI, %)		
Isometric	0.45	0.50
Isokinetic 60°s^−1^	**2.23**	**0.03**
Isokinetic 180°s^−1^	1.55	0.15
Flexor muscle strength (LSI, %)		
Isometric	−2.19	0.24
Isokinetic 60°s^−1^	0.88	0.11
Isokinetic 180°s^−1^	0.19	0.85
SLHT (LSI, %)	0.02	0.85
Graft rupture	0.00	0.82
Arthrofibrosis	0.00	0.70
Patellar fracture	0.13	1.00
Donor site morbidity	−0.07	0.11
VAS	−0.01	0.61

KOOS, knee injury and osteoarthritis outcome score; ADL, activities of daily living; IKDC, International Knee Documentation Committee score; SSD, side-to-side difference; LSI, limb symmetry index; SLHT, single leg hop test; VAS, visual analogue scale.

## Discussion

The most important finding in this study was that for primary ACLR, the QT autograft, whether it is harvested with or without a bone block, provides satisfactory functional outcomes, knee stability, and low complication rates. However, B-QT autografts appear to result in higher patient-reported outcomes, and S-QT autografts may result in slightly improved knee stability. Although a few systematic reviews on the topic of B-QT and S-QT autografts exist (8, 9), to the best of the authors’ knowledge, this is the first meta-analysis comparing B-QT and S-QT autografts in the setting of primary ACLR.

### Functional outcomes

Our first finding was that B-QT autografts appear to result in improved functional patient-reported outcomes. B-QT had significantly higher mean scores across all patient-reported outcome measures, including significantly higher scores for all five KOOS subscale domains (activities of daily living, pain, sports and recreation, symptoms, and quality of life) and significantly higher Marx scores than the S-QT subgroup. While differences between subgroups did not reach statistical significance for the IKDC subjective score, Lysholm and Tegner scores, the trend towards higher functional patient-reported outcomes in favour of B-QT was observed in each of those variables. This is in partial disagreement with a recent systematic review by Crum *et al.* (9); however, due to the lack of meta-analysis in their study, direct comparison is not possible. Importantly, the differences of mean KOOS subscales scores between B-QT and S-QT ranged from 2.10 (KOOS ADL), through 3.48 (KOOS pain), 5.05 (KOOS sports & recreation), to 6.56 (KOOS symptoms). None of these values exceeded the minimal clinically important difference (MCID), which, as most studies suggest, is somewhere in the 10-point range for the KOOS subscales (97).

### Objective outcomes

Our second finding was that S-QT autografts may result in slightly improved knee stability. The mean side-to-side difference for instrumented anterior knee laxity was significantly higher in the B-QT subgroup than in the S-QT subgroup. Although the differences did not reach statistical significance for the Lachman and pivot-shift tests, the trend towards lower knee stability in the B-QT group was present in these variables as well. This is in agreement with a recent systematic review by Crum *et al.* (9), who reported that a positive pivot-shift was found more often after B-QT than S-QT ACLR, suggesting that B-QT autografts may predispose patients to increased rotatory knee instability post-operatively (9, 23, 53, 75, 90). The explanation of this phenomenon is not known. However, it must be highlighted that the difference of mean SSD for anteroposterior tibial translation between B-QT and S-QT was only 0.4 mm. While statistically significant, those differences may not necessarily be clinically relevant. Further studies directly comparing stability between S-QT and B-QT are required.

### Muscle recovery

No differences in post-operative muscle strength recovery between the B-QT and S-QT groups were found in this study. A recent systematic review by Crum *et al.* described quadriceps strengths reported in the literature as ‘too heterogeneous to allow for relevant comparisons’ (9). We agree that high heterogeneity was present, i.e. in the follow-up times. However, in this study, due to the meta-analytical approach, the comparison was performed and 95% CIs were calculated to quantify the heterogeneity. The multivariate regression could also provide further insight into the impact of reported follow-up length on the muscle strength recovery.

### Complications

As to the complications, a recent systematic review comparing the incidences of complications after primary ACLR between B-QT and S-QT autografts found that patients who underwent ACLR with S-QT grafts had a 2.7-times increased incidence of anterior knee pain (23.3 vs 8.6%) (8). This is in contrast to the results of our study, with a prevalence of donor site morbidity – defined primarily as anterior knee pain – of 3.7% for the S-QT group and 6.6% for the B-QT group. Although the authors did not specify the exact statistical method by which the incidences were synthesised across the studies included in their study, it is likely that they were calculated by simple pooling. Simple pooling, by treating the data of individual studies as if it came from one large trial, has been shown to not be as accurate as combining by meta-analytic methods in deriving a summary estimate that deals with proportions (98).

### Limitations

As with each literature review, this study was limited by the literature availability. The first limitation was a relatively low percentage of randomised controlled trials (thirteen studies out of 92 included in the meta-analysis). The second limitation was the relatively low methodological quality of included studies, both in randomised controlled trials (six with ‘some concerns’ of bias, seven at ‘high risk’ of bias), and non-randomised studies: cohort studies (median MINORS 10/16) and comparative studies (median MINORS 17/24). Methodological limitations could influence the results both in favour of B-QT and S-QT, limiting the strength of the conclusions. This highlights the need for further high-quality, prospective randomised trials directly comparing B-QT and S-QT autografts. The third limitation was the mismatch between the number of patients in the B-QT and S-QT groups (5,898 B-QT patients vs 1,849 S-QT patients). We acknowledge that the considerable imbalance in sample sizes may have influenced the statistical power of comparisons, potentially favouring results in the larger B-QT cohort. To provide the valuable meta-analysis despite limitations, special care and attention were placed during every step of the research synthesis, in order to ensure accuracy, transparency, and reproducibility. Furthermore, the data were extracted while adhering to strict protocols and following validated protocols for data extractions, such as the creation and piloting of a custom, standardised data collection form, and the use of a relational database with adequately normalised database tables.

### Clinically oriented summary for graft selection

The differences between B-QT and S-QT were relatively small. Functional outcomes differences between B-QT and S-QT did not exceed MCID. The difference of mean SSD for anteroposterior tibial translation between B-QT and S-QT was only 0.4 mm. While statistically significant, those differences may not necessarily be clinically relevant. Both B-QT and S-QT were safe and effective. Therefore, we feel that the data from this study reassure usage of both types of QT autograft, rather than guiding the selection between B-QT and S-QT. Surgeon experience and patient-specific factors should guide graft choice depending on the specific scenarios. For example, B-QT may offer longer graft, bone-to-bone healing, and possibility to use in revision cases with bone deficiency. On the other hand, harvesting S-QT is less challenging technically and yields no risk of patellar fracture.

## Conclusion

A QT autograft, with or without a bone block, provides satisfactory functional outcomes, knee stability, and low complications in primary ACLR. B-QT may result in higher patient-reported outcomes, while S-QT may potentially result in slightly improved knee stability.

### Summary points

- Both quadriceps tendon autografts (B-QT and S-QT) are reliable options for primary ACL reconstruction, providing satisfactory functional outcomes, stability, and low complication rates.
- B-QT may result in higher patient-reported outcomes, while S-QT may potentially result in slightly improved knee stability. Those differences are small, with questionable clinical importance.
- No significant differences in postoperative muscle strength recovery were observed between B-QT and S-QT groups.
- Quality of included evidence remains limited due to relatively few high-quality RCTs and methodological variability.

## Supplementary materials



## ICMJE Statement of Interest

The authors declare that there is no conflict of interest that could be perceived as prejudicing the impartiality of the work reported.

## Funding Statement

This work did not receive any specific grant from any funding agency in the public, commercial, or not-for-profit sector.

## Author contribution statement

All authors contributed to the study conception and design. Material preparation, data collection, and analysis were performed by KM, DWK, JS, ZM, LT, and RFL. The first draft of the manuscript was written by DWK and MM, and all authors commented on previous versions of the manuscript. All authors read and approved the final manuscript.

## Data availability

On request, on OSF.io, and on github.com.
