# Supplementary material for: Quadriceps tendon-bone vs all soft-tissue autografts for primary anterior cruciate ligament reconstruction: a systematic review and meta-analysis of 7,748 patients
Source: EFORT Open Rev. 2025 Dec 5;10(12):865–81. doi: 10.1530/EOR-2024-0204 (PMC12687117; doi:10.1530/EOR-2024-0204)

Supplementary File

Supplementary Figure 1 Forest plot of IKDC subjective scores between S-QT and B-QT subgroups

Author(s) and Year	N	Mean	SD	Mean [95% CI]
**S-QT**				
Brinkman et al., 2023	37	90.70	6.00	90.70 [88.77, 92.63]
DeAngelis et al., 2007	124	84.70	15.65	84.70 [81.95, 87.45]
Gille et al., 2009	54	78.30	14.63	78.30 [74.40, 82.20]
Greif et al., 2022	124	91.10	11.15	91.10 [89.14, 93.06]
Hogan et al., 2022	19	77.40	24.60	77.40 [66.34, 88.46]
Hunnicutt et al., 2019	15	81.29	7.82	81.29 [77.33, 85.25]
Johnston et al., 2021	36	77.71	14.33	77.71 [73.03, 82.39]
Johnston et al., 2022	32	88.50	11.90	88.50 [84.38, 92.62]
Letter et al., 2019	17	71.71	12.41	71.71 [65.81, 77.61]
Letter et al., 2023	26	69.42	11.53	69.42 [64.99, 73.85]
Perez et al., 2019	28	94.83	5.94	94.83 [92.63, 97.03]
Pichler et al., 2022	40	93.90	13.91	93.90 [89.59, 98.21]
Renfree et al., 2023	32	90.50	6.60	90.50 [88.21, 92.79]
Schulz et al., 2013	55	80.44	12.05	80.44 [77.26, 83.62]
Setliff et al., 2023.2	120	81.00	18.90	81.00 [77.62, 84.38]
Tang et al., 2023	17	87.12	6.61	87.12 [83.98, 90.26]
RE Model for Subgroup: $\tau^2 = 53.95$, $I^2 = 95.6\%$, $p < 0.001$				83.97 [80.23, 87.72]
**B-QT**				
Akoto and Hoeher, 2012	30	86.10	15.80	86.10 [80.45, 91.75]
Akoto et al., 2015	10	89.50	8.30	89.50 [84.36, 94.64]
Akoto et al., 2019	41	86.40	14.20	86.40 [82.05, 90.75]
Barié et al., 2010	106	83.78	13.32	83.78 [81.24, 86.32]
Barié et al., 2018	57	90.00	9.90	90.00 [87.43, 92.57]
Barié et al., 2020	21	92.00	11.50	92.00 [87.08, 96.92]
Cavaignac et al., 2017	44	84.00	13.00	84.00 [80.16, 87.84]
Fu et al., 2019	51	89.80	10.61	89.80 [86.89, 92.71]
Guney-Deniz et al., 2020	22	82.70	9.10	82.70 [78.90, 86.50]
Herman et al., 2023	14	91.60	9.70	91.60 [86.52, 96.68]
Hofer et al., 2022	27	88.00	12.40	88.00 [83.32, 92.68]
Horstmann et al., 2022	24	89.30	12.20	89.30 [84.42, 94.18]
Irrgang et al., 2021	51	92.14	4.46	92.14 [90.92, 93.36]
Kim et al., 2014	142	87.02	6.84	87.02 [85.89, 88.15]
Kim et al., 2018	75	71.07	9.55	71.07 [68.91, 73.23]
Komzák et al., 2022	40	78.30	10.50	78.30 [75.05, 81.55]
Kwak et al., 2018	45	67.30	16.80	67.30 [62.39, 72.21]
Lee et al., 2014	104	77.25	13.49	77.25 [74.66, 79.84]
Lee et al., 2016	48	80.20	10.00	80.20 [77.37, 83.03]
Lee et al., 2021	139	81.00	8.40	81.00 [79.60, 82.40]
Lind et al., 2020	47	82.00	14.00	82.00 [78.00, 86.00]
Lund et al., 2014	21	84.00	13.00	84.00 [78.44, 89.56]
Pomenta Bastidas et al., 2022	20	86.28	6.80	86.28 [83.30, 89.26]
Runer et al., 2023	28	93.90	6.80	93.90 [91.38, 96.42]
Setliff et al., 2023.1	10	80.90	20.40	80.90 [68.26, 93.54]
Sinding et al., 2020	42	76.00	17.00	76.00 [70.86, 81.14]
Vilchez-Cavazos et al., 2020	14	88.00	7.00	88.00 [84.33, 91.67]
RE Model for Subgroup: $\tau^2 = 38.64$, $I^2 = 95.3\%$, $p < 0.001$				84.45 [81.97, 86.92]
RE Model for All Studies: $\tau^2 = 42.94$, $I^2 = 95.4\%$, $p < 0.001$				84.29 [82.23, 86.35]
Test for Subgroup Differences: $Q_M = 0.04$, df = 1, $p = 0.837$				

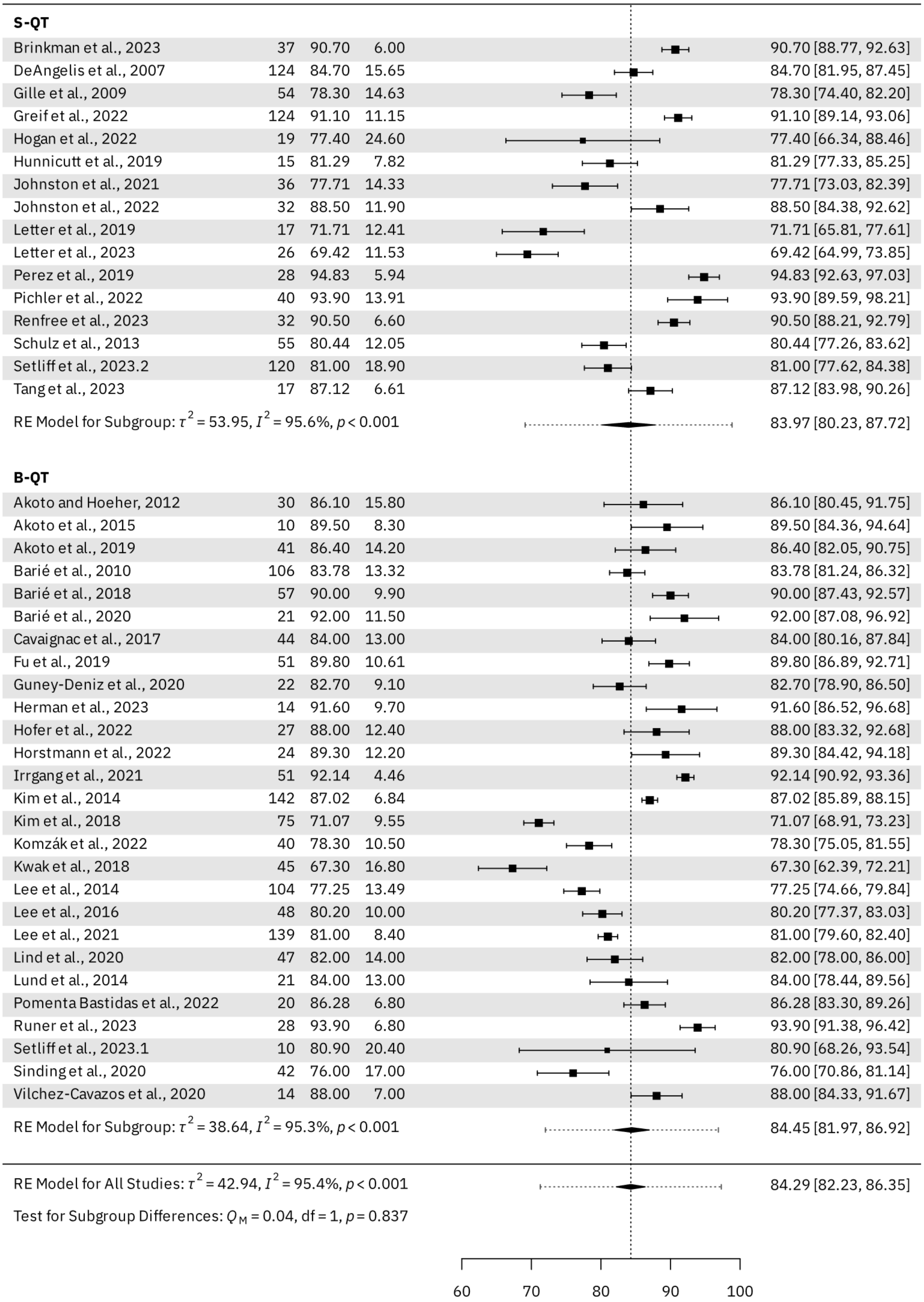

Supplementary File

Supplementary Figure 2 Forest plot of Lysholm scores between S-QT and B-QT subgroups

Author(s) and Year	N	Mean	SD	Mean [95% CI]
**S-QT**				
Aslam et al., 2021	35	86.04	3.10	86.04 [85.01, 87.07]
Brinkman et al., 2023	37	92.60	5.30	92.60 [90.89, 94.31]
Gille et al., 2009	54	80.80	17.60	80.80 [76.11, 85.49]
Greif et al., 2022	124	92.11	9.11	92.11 [90.51, 93.71]
Hunnicutt et al., 2019	15	85.86	7.19	85.86 [82.22, 89.50]
Letter et al., 2019	17	82.12	13.24	82.12 [75.83, 88.41]
Letter et al., 2023	26	90.15	11.79	90.15 [85.62, 94.68]
Perez et al., 2019	28	95.00	7.03	95.00 [92.40, 97.60]
Pichler et al., 2022	40	95.50	11.59	95.50 [91.91, 99.09]
Renfree et al., 2023	32	91.30	7.50	91.30 [88.70, 93.90]
Schagemann et al., 2021	8	86.88	10.09	86.88 [79.89, 93.87]
Schulz et al., 2013	55	89.00	17.70	89.00 [84.32, 93.68]
Tang et al., 2023	17	90.00	4.26	90.00 [87.97, 92.03]
Todor et al., 2019	39	89.20	9.97	89.20 [86.07, 92.33]
RE Model for Subgroup: $\tau^2 = 13.67$, $I^2 = 89.8\%$, $p < 0.001$				89.41 [87.25, 91.58]
**B-QT**				
Barié et al., 2010	105	88.50	12.70	88.50 [86.07, 90.93]
Barié et al., 2018	57	92.00	10.60	92.00 [89.25, 94.75]
Barié et al., 2020	21	95.90	6.10	95.90 [93.29, 98.51]
Cavaignac et al., 2017	44	89.00	6.90	89.00 [86.96, 91.04]
Chen et al., 2006	34	93.00	7.90	93.00 [90.34, 95.66]
Galan et al., 2020	291	91.00	6.05	91.00 [90.30, 91.70]
Gorschewsky et al., 2007a	193	91.41	10.65	91.41 [89.91, 92.91]
Gorschewsky et al., 2007b	93	94.00	9.00	94.00 [92.17, 95.83]
Guimarães et al., 2009	17	97.35	4.11	97.35 [95.40, 99.30]
Hofer et al., 2022	27	91.00	9.00	91.00 [87.61, 94.39]
Horstmann et al., 2022	24	90.40	11.90	90.40 [85.64, 95.16]
Jacquet et al., 2021	91	90.10	13.30	90.10 [87.37, 92.83]
Karpinski et al., 2021	25	88.60	11.78	88.60 [83.98, 93.22]
Kim et al., 2009c	29	91.10	6.80	91.10 [88.63, 93.57]
Kim et al., 2009d	59	93.22	8.83	93.22 [90.97, 95.47]
Kim et al., 2014	142	88.10	6.43	88.10 [87.04, 89.16]
Kim et al., 2018	75	89.75	8.76	89.75 [87.77, 91.73]
Komzák et al., 2022	40	90.10	6.70	90.10 [88.02, 92.18]
Kuangyang et al., 2022	33	89.39	5.33	89.39 [87.57, 91.21]
Kwak et al., 2018	45	87.00	5.30	87.00 [85.45, 88.55]
Lee et al., 2007a	112	92.57	9.37	92.57 [90.83, 94.31]
Lee et al., 2014	104	90.60	8.50	90.60 [88.97, 92.23]
Lee et al., 2016	48	92.10	8.70	92.10 [89.64, 94.56]
Lee et al., 2021	139	89.80	8.30	89.80 [88.42, 91.18]
Mouarbes et al., 2020	30	90.10	10.10	90.10 [86.49, 93.71]
Mutsuzaki et al., 2019	7	94.40	4.40	94.40 [91.14, 97.66]
Pomenta Bastidas et al., 2022	20	96.05	2.17	96.05 [95.10, 97.00]
Runer et al., 2018	40	93.40	7.50	93.40 [91.08, 95.72]
Runer et al., 2020	114	86.00	22.30	86.00 [81.91, 90.09]
Runer et al., 2023	28	91.90	7.20	91.90 [89.23, 94.57]
Vilchez-Cavazos et al., 2020	14	95.36	7.41	95.36 [91.48, 99.24]
Yamasaki et al., 2023	21	92.89	15.11	92.89 [86.43, 99.35]
RE Model for Subgroup: $\tau^2 = 5.76$, $I^2 = 86.6\%$, $p < 0.001$				91.45 [90.51, 92.40]
RE Model for All Studies: $\tau^2 = 7.67$, $I^2 = 88.5\%$, $p < 0.001$				90.93 [90.03, 91.84]
Test for Subgroup Differences: $Q_M = 2.79$, df = 1, $p = 0.095$				

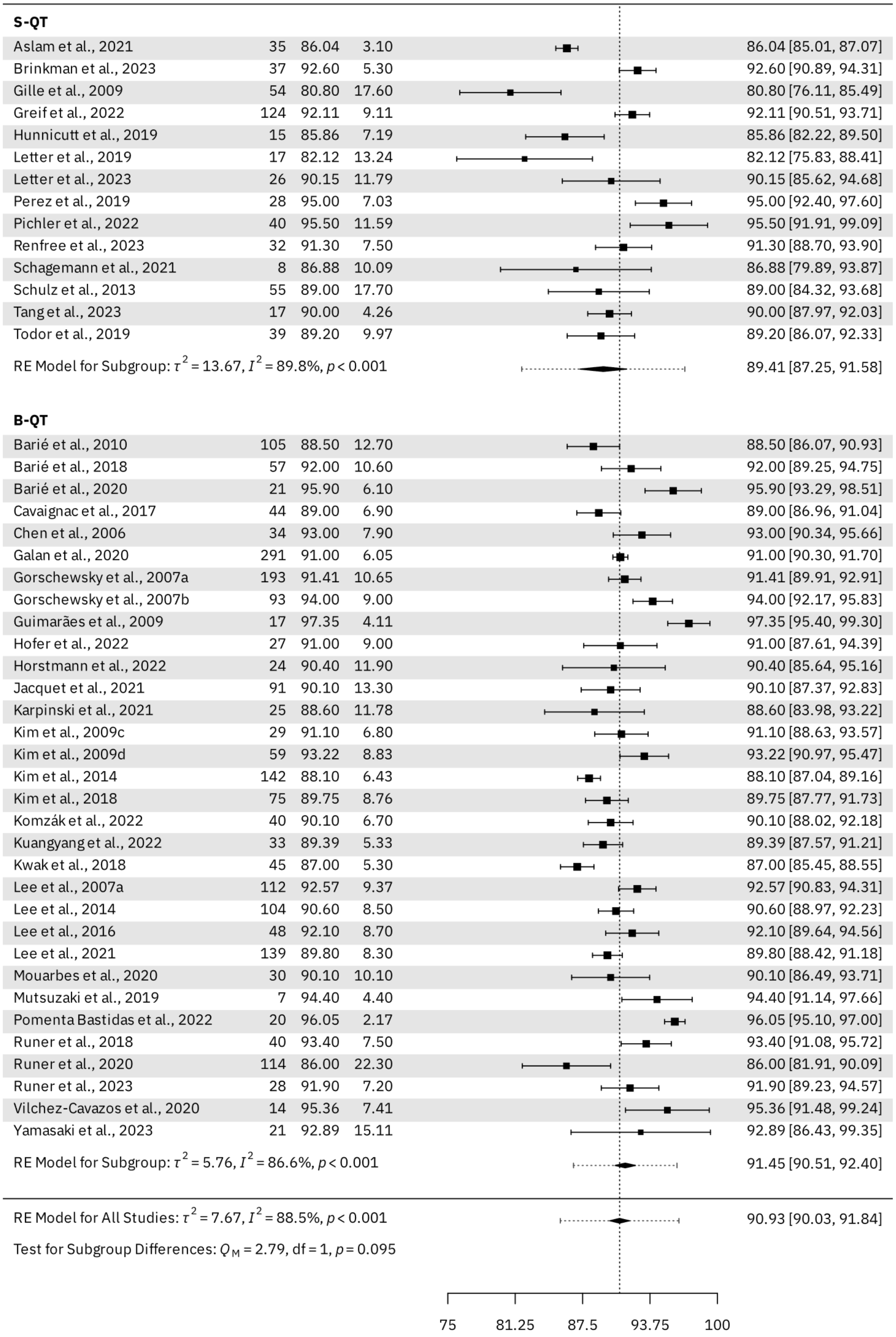

Supplementary File

Supplementary Figure 3 Forest plot of Tegner scores for the B-QT and S-QT subgroups

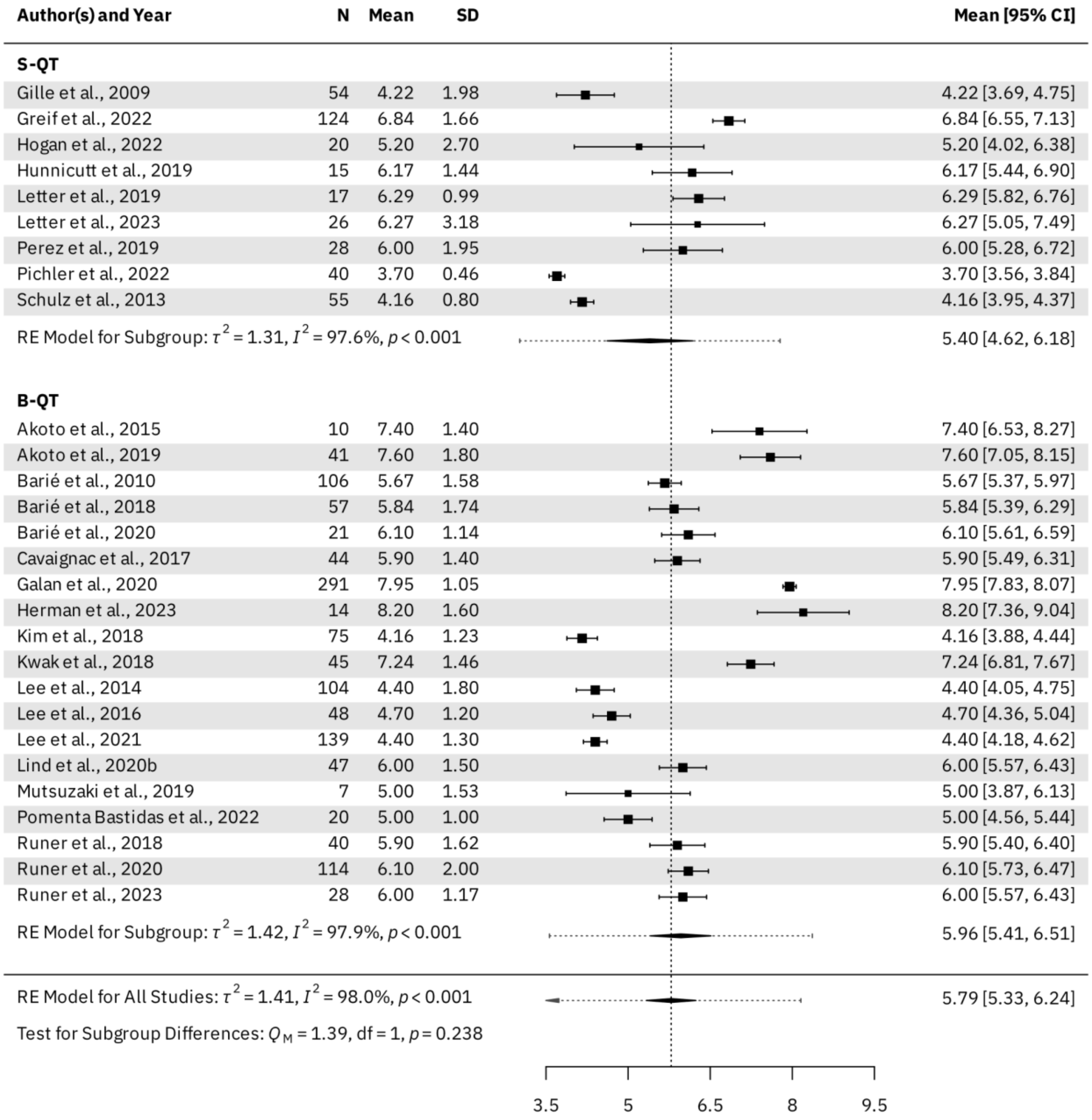

Supplementary File

Supplementary Figure 4 Forest plot of IKDC objective scores for the B-QT and S-QT subgroups

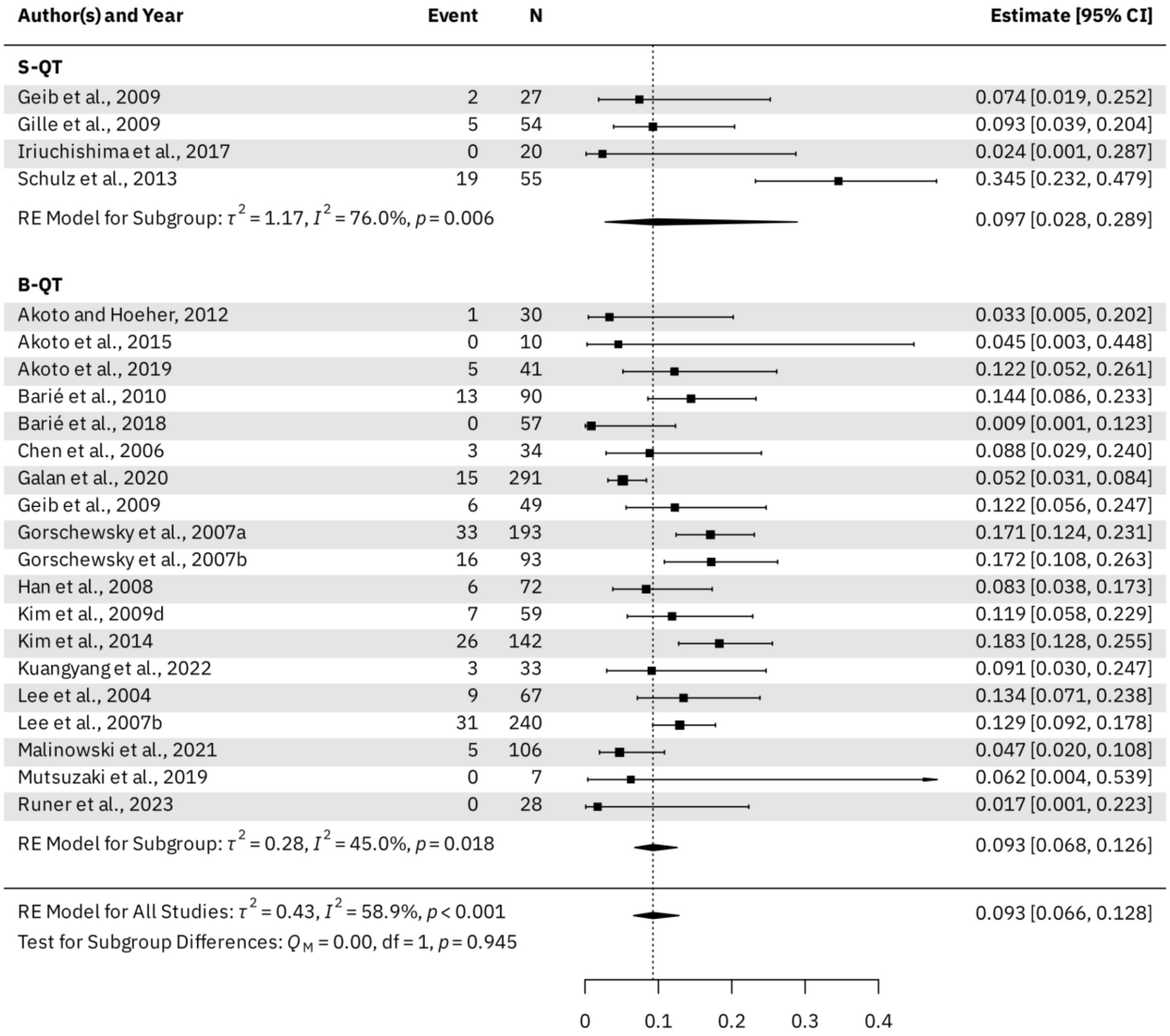

Supplementary File

Supplmentary Figure 5 Forest plot of Lachman for the B-QT and S-QT subgroups

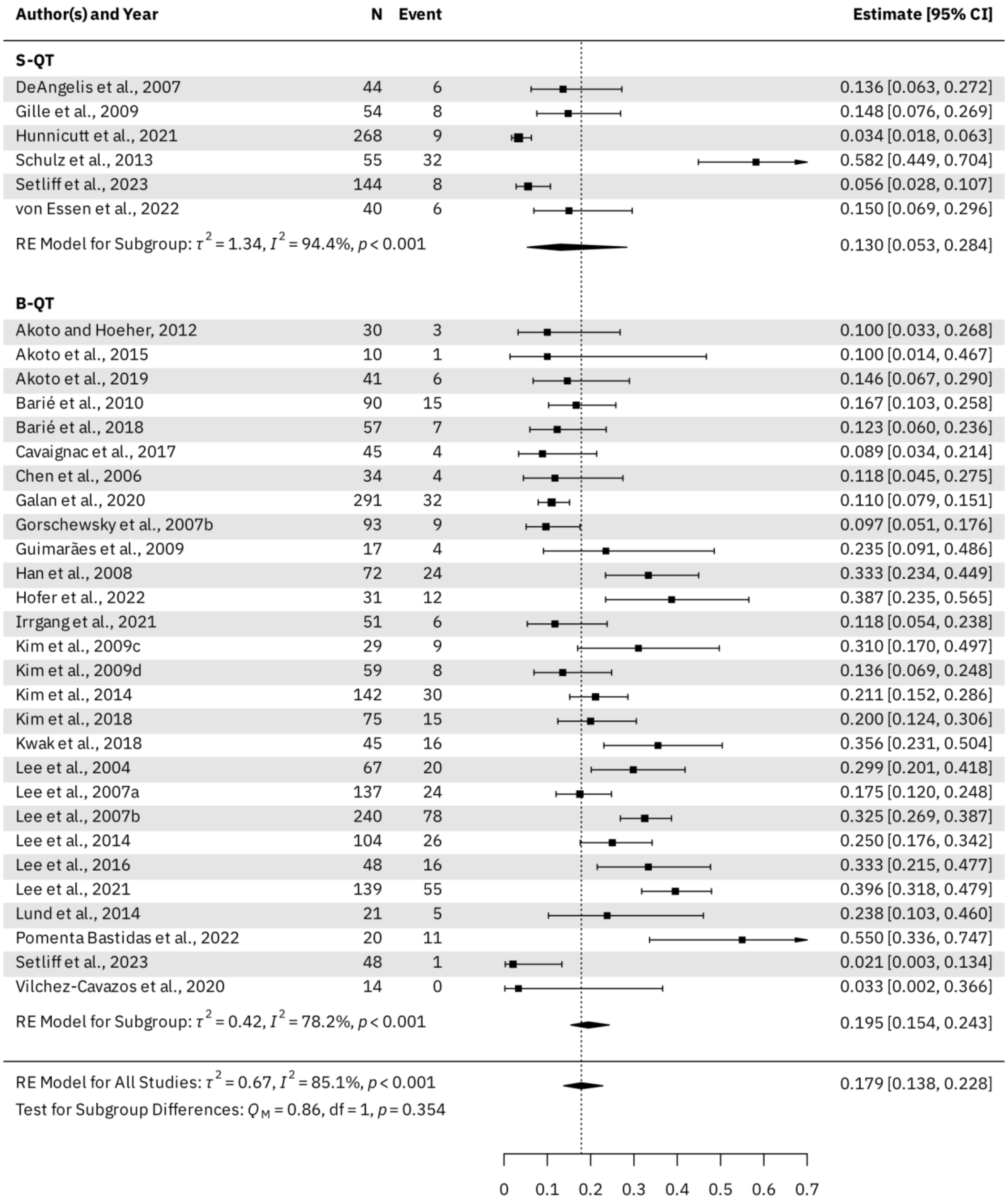

Supplementary File

Supplementary Figure 6 Forest plot of pivot shift for the B-QT and S-QT subgroups

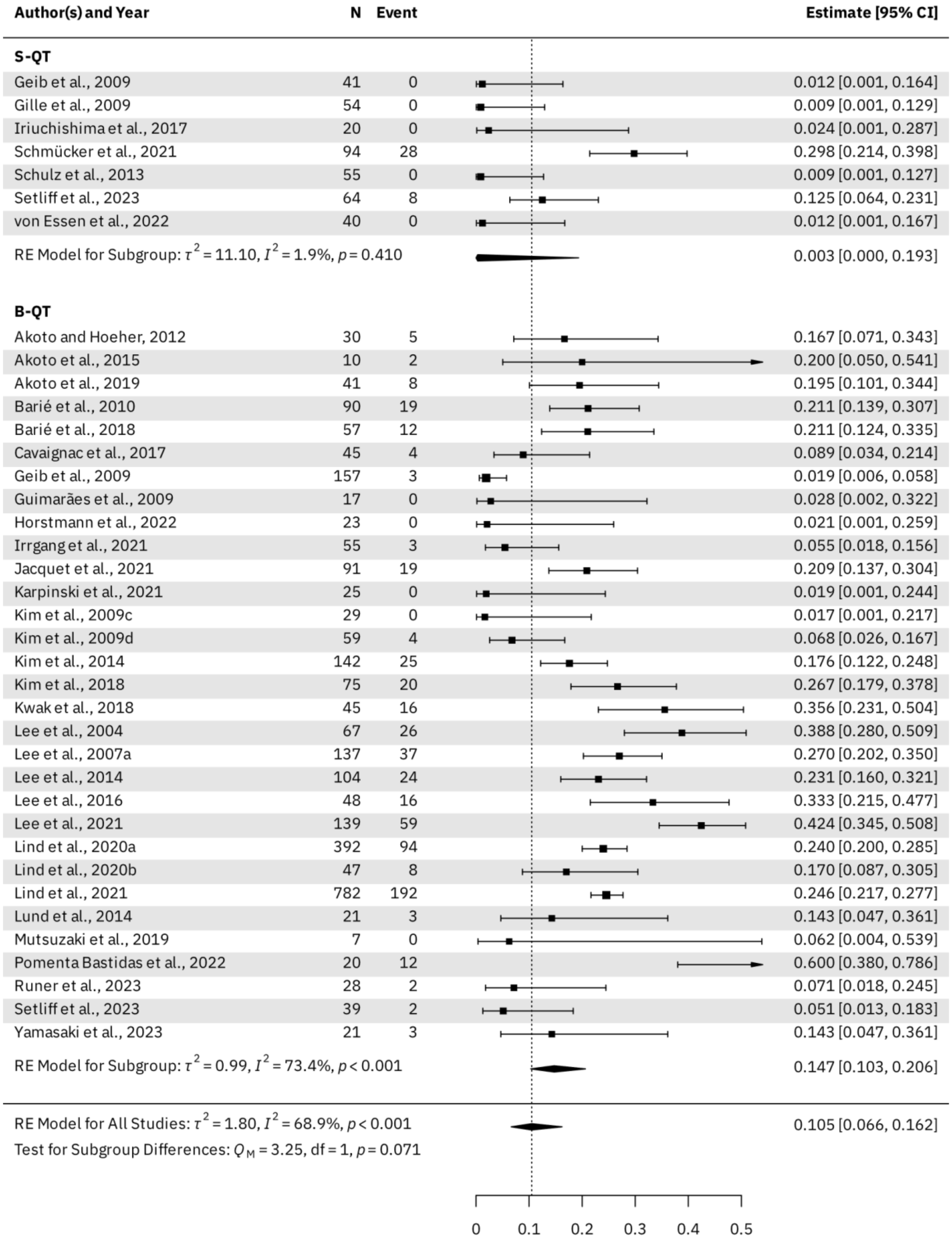

Supplementary File

Supplementary Figure 7 Forest plot of isometric peak extensor torque, LSI (%) for the B-QT and S-QT subgroups

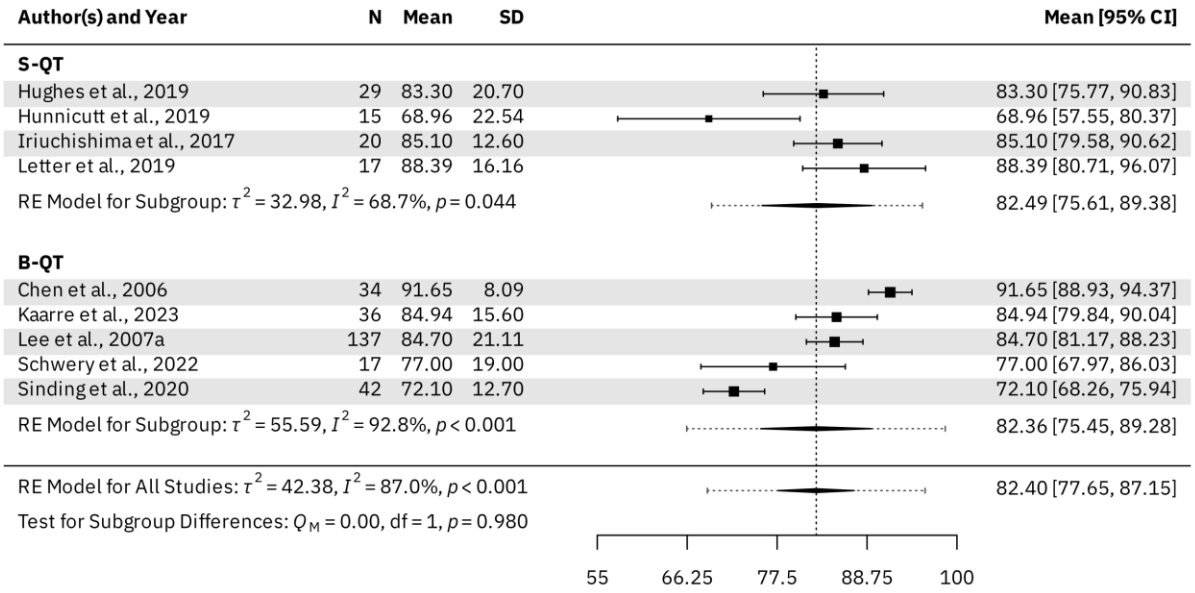

Supplementary File

Supplementary Figure 8 Forest plot of isokinetic peak extensor torque (60°/s), LSI (%) for the B-QT and S-QT subgroups

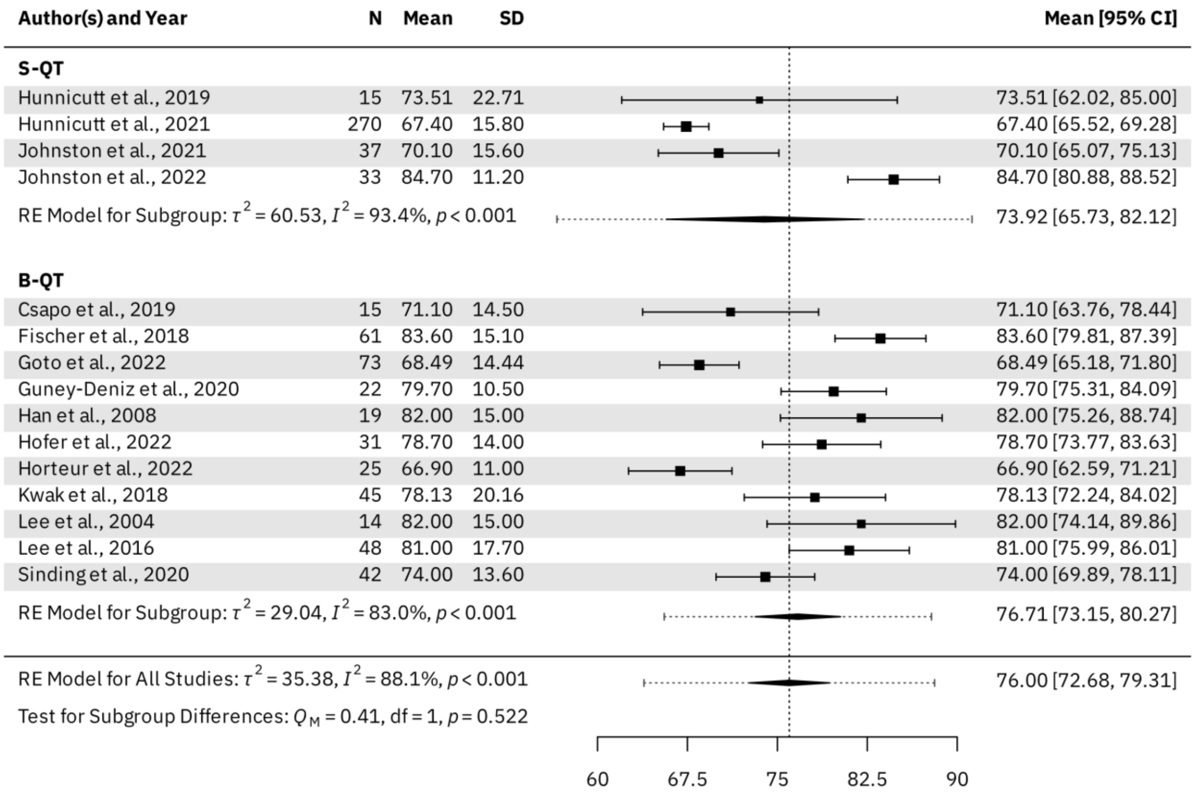

Supplementary File

Supplementary Figure 9 Forest plot of isokinetic peak extensor torque (180°/s), LSI (%) for the B-QT and S-QT subgroups

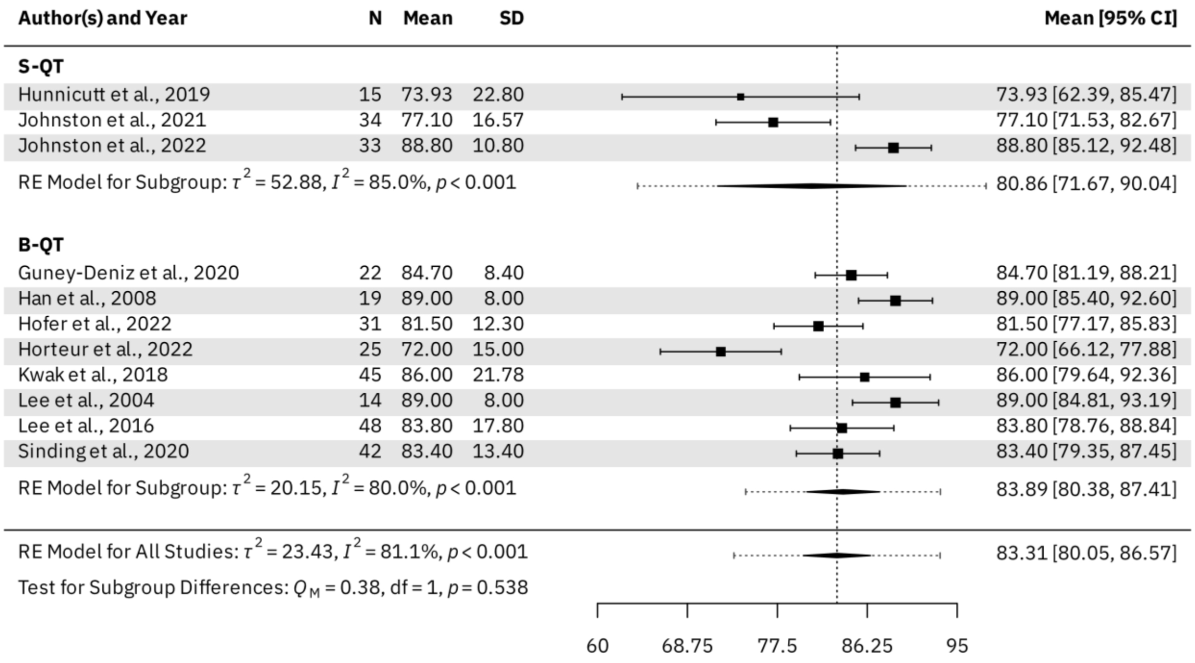

Supplementary File

Supplementary Figure 10 Forest plots of isometric peak flexor torque LSI (%) for the B-QT and S-QT subgroups

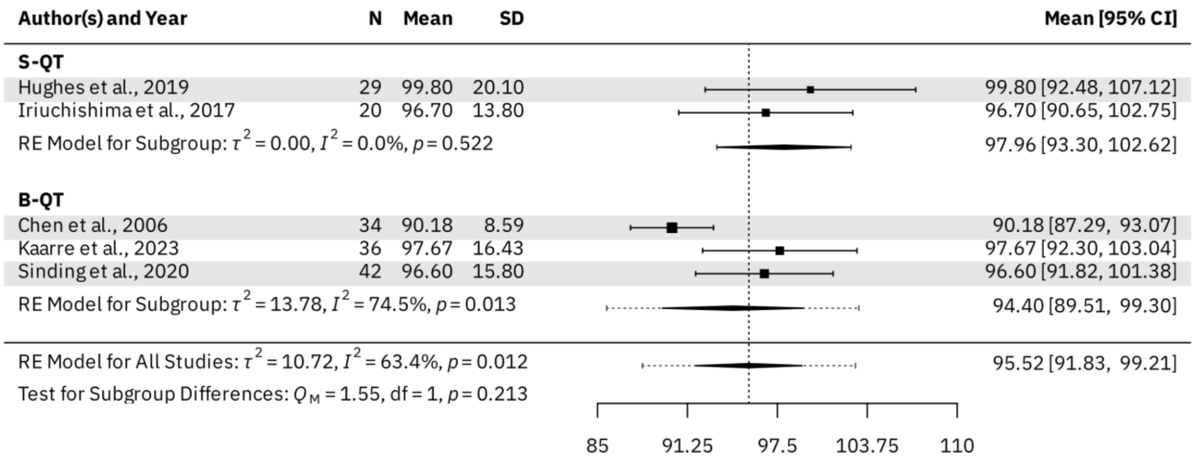

Supplementary File

Supplementary Figure 11 Forest plots of isokinetic peak flexor torque (60°/s), LSI (%) for the B-QT and S-QT subgroups

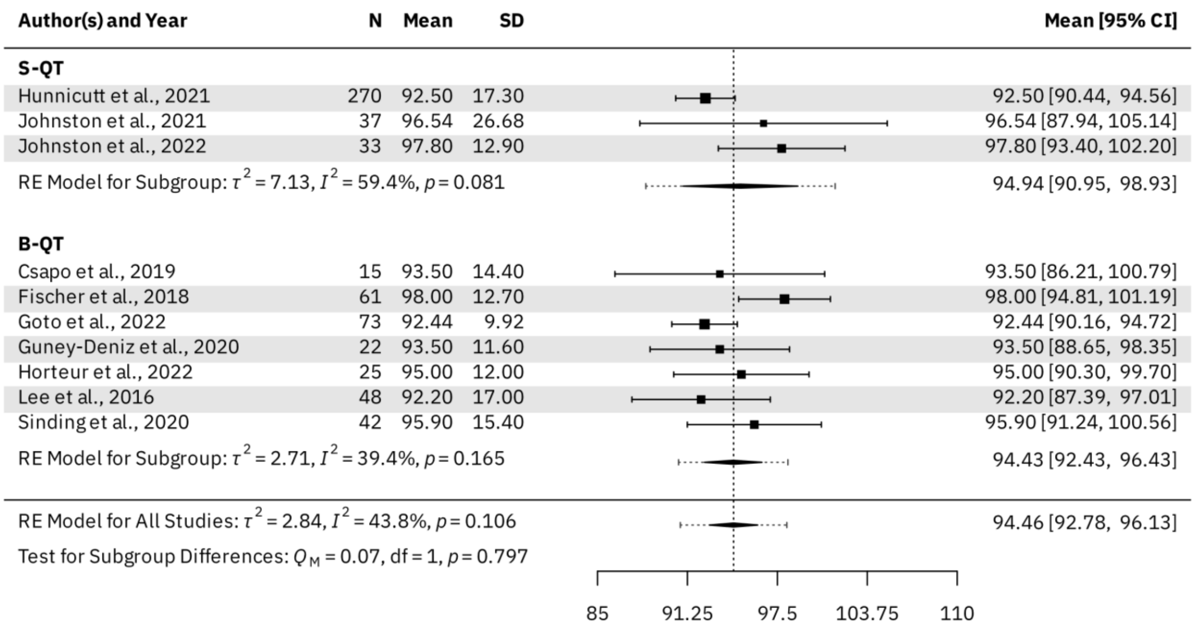

Supplementary File

Supplementary Figure 12 Forest plots of isokinetic peak flexor torque (180°/s), LSI (%) for the B-QT and S-QT subgroups

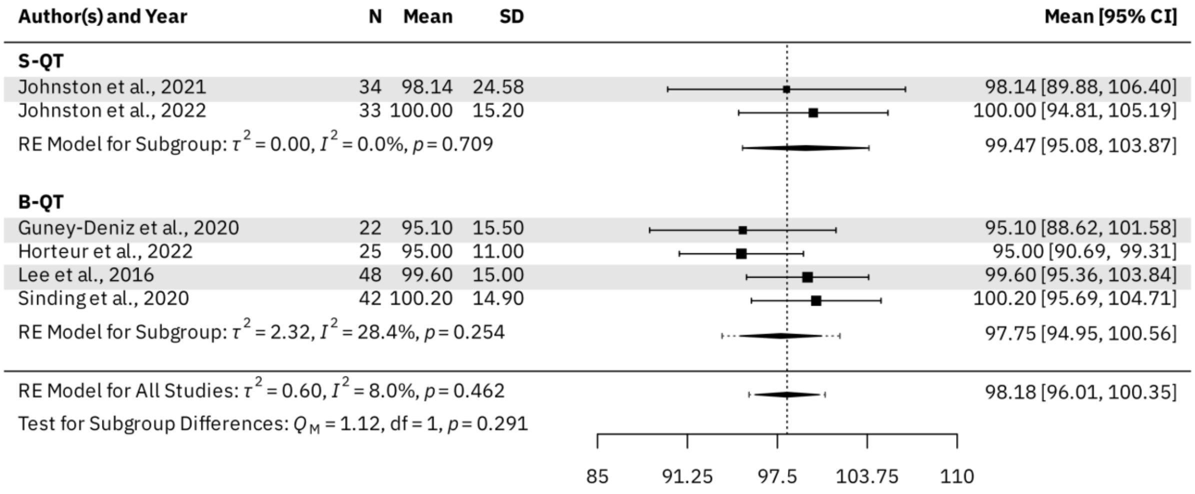

Supplementary File

Supplementary Figure 13 Forest plots of single leg triple hop test (SLHT) for the B-QT and S-QT subgroups

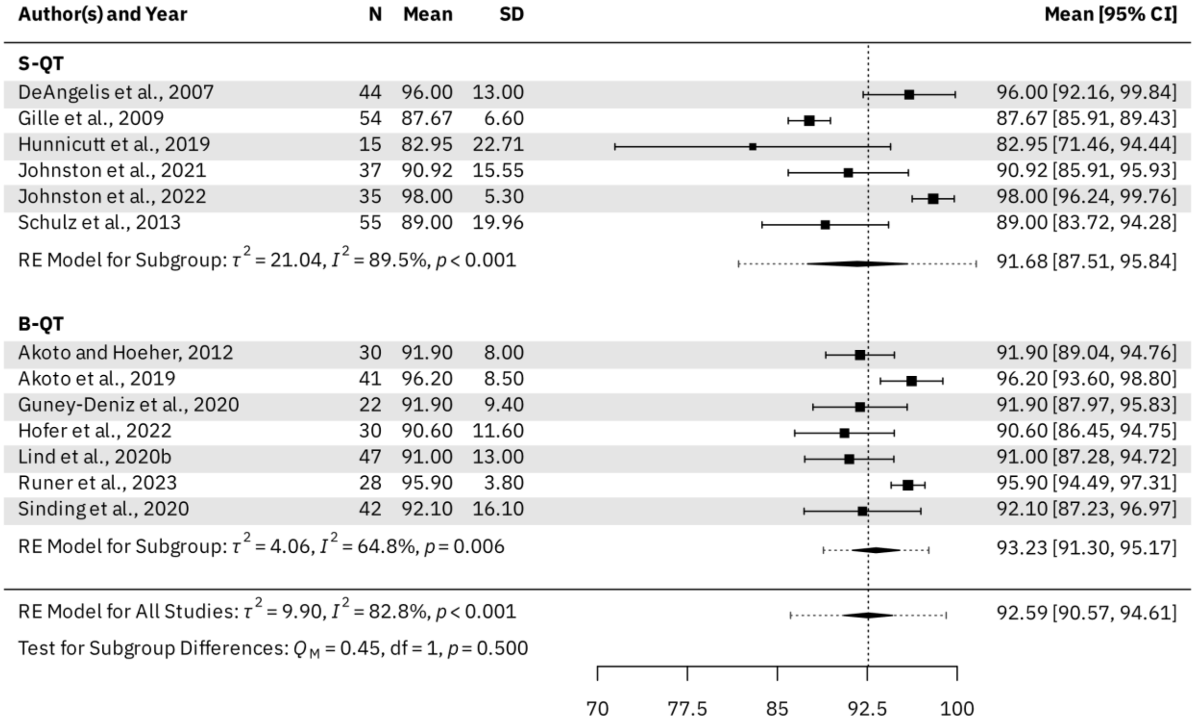

Supplementary Figure 14 Forest plots of graft rupture for the B-QT and S-QT subgroups

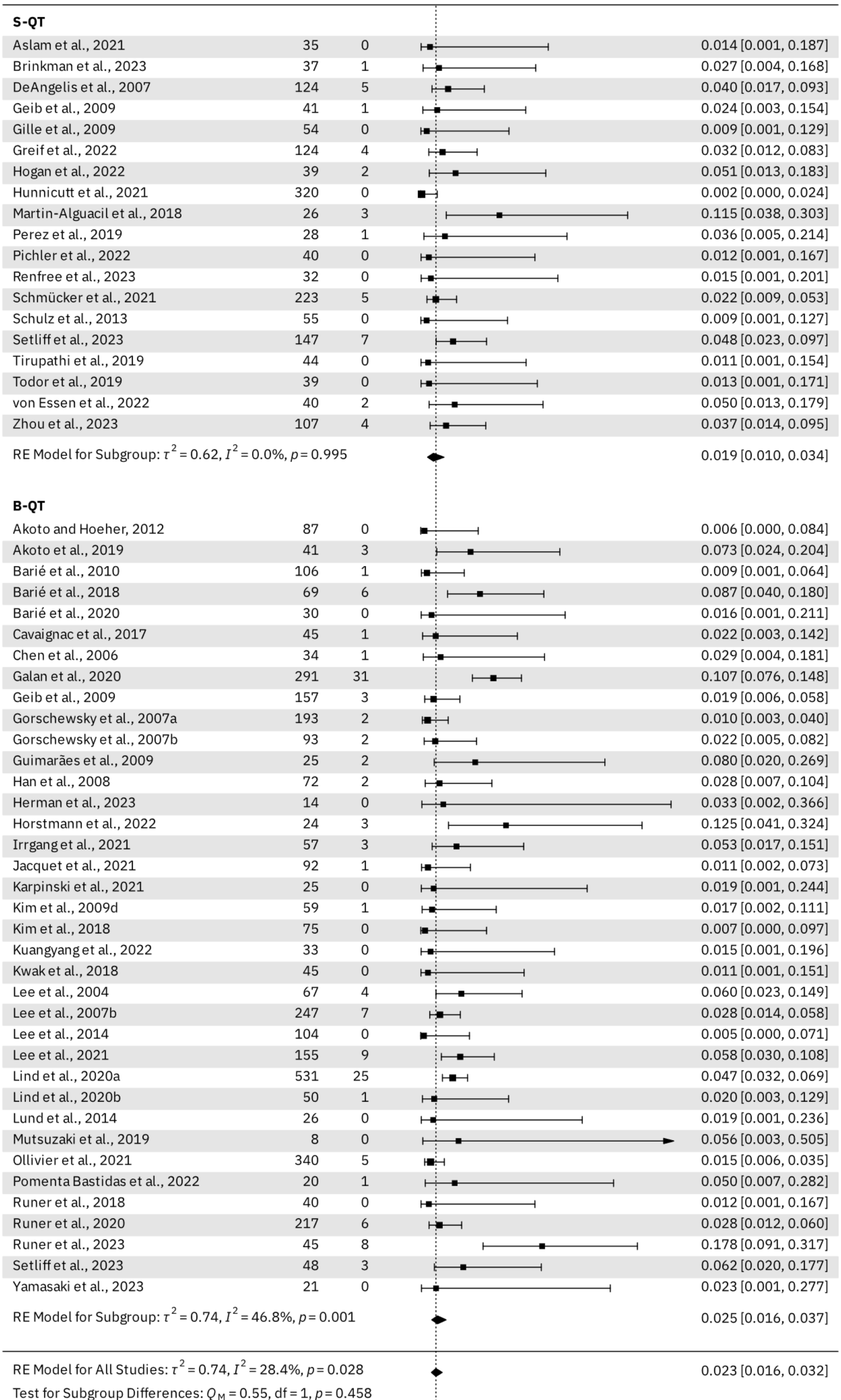

Author(s) and Year	N	Event	Estimate [95% CI]
**S-QT**			
Aslam et al., 2021	35	0	0.014 [0.001, 0.187]
Brinkman et al., 2023	37	1	0.027 [0.004, 0.168]
DeAngelis et al., 2007	124	5	0.040 [0.017, 0.093]
Geib et al., 2009	41	1	0.024 [0.003, 0.154]
Gille et al., 2009	54	0	0.009 [0.001, 0.129]
Greif et al., 2022	124	4	0.032 [0.012, 0.083]
Hogan et al., 2022	39	2	0.051 [0.013, 0.183]
Hunnicutt et al., 2021	320	0	0.002 [0.000, 0.024]
Martin-Alguacil et al., 2018	26	3	0.115 [0.038, 0.303]
Perez et al., 2019	28	1	0.036 [0.005, 0.214]
Pichler et al., 2022	40	0	0.012 [0.001, 0.167]
Renfree et al., 2023	32	0	0.015 [0.001, 0.201]
Schmücker et al., 2021	223	5	0.022 [0.009, 0.053]
Schulz et al., 2013	55	0	0.009 [0.001, 0.127]
Setliff et al., 2023	147	7	0.048 [0.023, 0.097]
Tirupathi et al., 2019	44	0	0.011 [0.001, 0.154]
Todor et al., 2019	39	0	0.013 [0.001, 0.171]
von Essen et al., 2022	40	2	0.050 [0.013, 0.179]
Zhou et al., 2023	107	4	0.037 [0.014, 0.095]
RE Model for Subgroup: $\tau^2 = 0.62$, $I^2 = 0.0\%$, $p = 0.995$			0.019 [0.010, 0.034]
**B-QT**			
Akoto and Hoeher, 2012	87	0	0.006 [0.000, 0.084]
Akoto et al., 2019	41	3	0.073 [0.024, 0.204]
Barié et al., 2010	106	1	0.009 [0.001, 0.064]
Barié et al., 2018	69	6	0.087 [0.040, 0.180]
Barié et al., 2020	30	0	0.016 [0.001, 0.211]
Cavaignac et al., 2017	45	1	0.022 [0.003, 0.142]
Chen et al., 2006	34	1	0.029 [0.004, 0.181]
Galan et al., 2020	291	31	0.107 [0.076, 0.148]
Geib et al., 2009	157	3	0.019 [0.006, 0.058]
Gorschewsky et al., 2007a	193	2	0.010 [0.003, 0.040]
Gorschewsky et al., 2007b	93	2	0.022 [0.005, 0.082]
Guimarães et al., 2009	25	2	0.080 [0.020, 0.269]
Han et al., 2008	72	2	0.028 [0.007, 0.104]
Herman et al., 2023	14	0	0.033 [0.002, 0.366]
Horstmann et al., 2022	24	3	0.125 [0.041, 0.324]
Irrgang et al., 2021	57	3	0.053 [0.017, 0.151]
Jacquet et al., 2021	92	1	0.011 [0.002, 0.073]
Karpinski et al., 2021	25	0	0.019 [0.001, 0.244]
Kim et al., 2009d	59	1	0.017 [0.002, 0.111]
Kim et al., 2018	75	0	0.007 [0.000, 0.097]
Kuangyang et al., 2022	33	0	0.015 [0.001, 0.196]
Kwak et al., 2018	45	0	0.011 [0.001, 0.151]
Lee et al., 2004	67	4	0.060 [0.023, 0.149]
Lee et al., 2007b	247	7	0.028 [0.014, 0.058]
Lee et al., 2014	104	0	0.005 [0.000, 0.071]
Lee et al., 2021	155	9	0.058 [0.030, 0.108]
Lind et al., 2020a	531	25	0.047 [0.032, 0.069]
Lind et al., 2020b	50	1	0.020 [0.003, 0.129]
Lund et al., 2014	26	0	0.019 [0.001, 0.236]
Mutsuzaki et al., 2019	8	0	0.056 [0.003, 0.505]
Ollivier et al., 2021	340	5	0.015 [0.006, 0.035]
Pomenta Bastidas et al., 2022	20	1	0.050 [0.007, 0.282]
Runer et al., 2018	40	0	0.012 [0.001, 0.167]
Runer et al., 2020	217	6	0.028 [0.012, 0.060]
Runer et al., 2023	45	8	0.178 [0.091, 0.317]
Setliff et al., 2023	48	3	0.062 [0.020, 0.177]
Yamasaki et al., 2023	21	0	0.023 [0.001, 0.277]
RE Model for Subgroup: $\tau^2 = 0.74$, $I^2 = 46.8\%$, $p = 0.001$			0.025 [0.016, 0.037]
RE Model for All Studies: $\tau^2 = 0.74$, $I^2 = 28.4\%$, $p = 0.028$			0.023 [0.016, 0.032]
Test for Subgroup Differences: $Q_M = 0.55$, df = 1, $p = 0.458$			

Supplementary File

Supplementary Figure 15 Forest plot of arthrofibrosis for the B-QT and S-QT subgroups

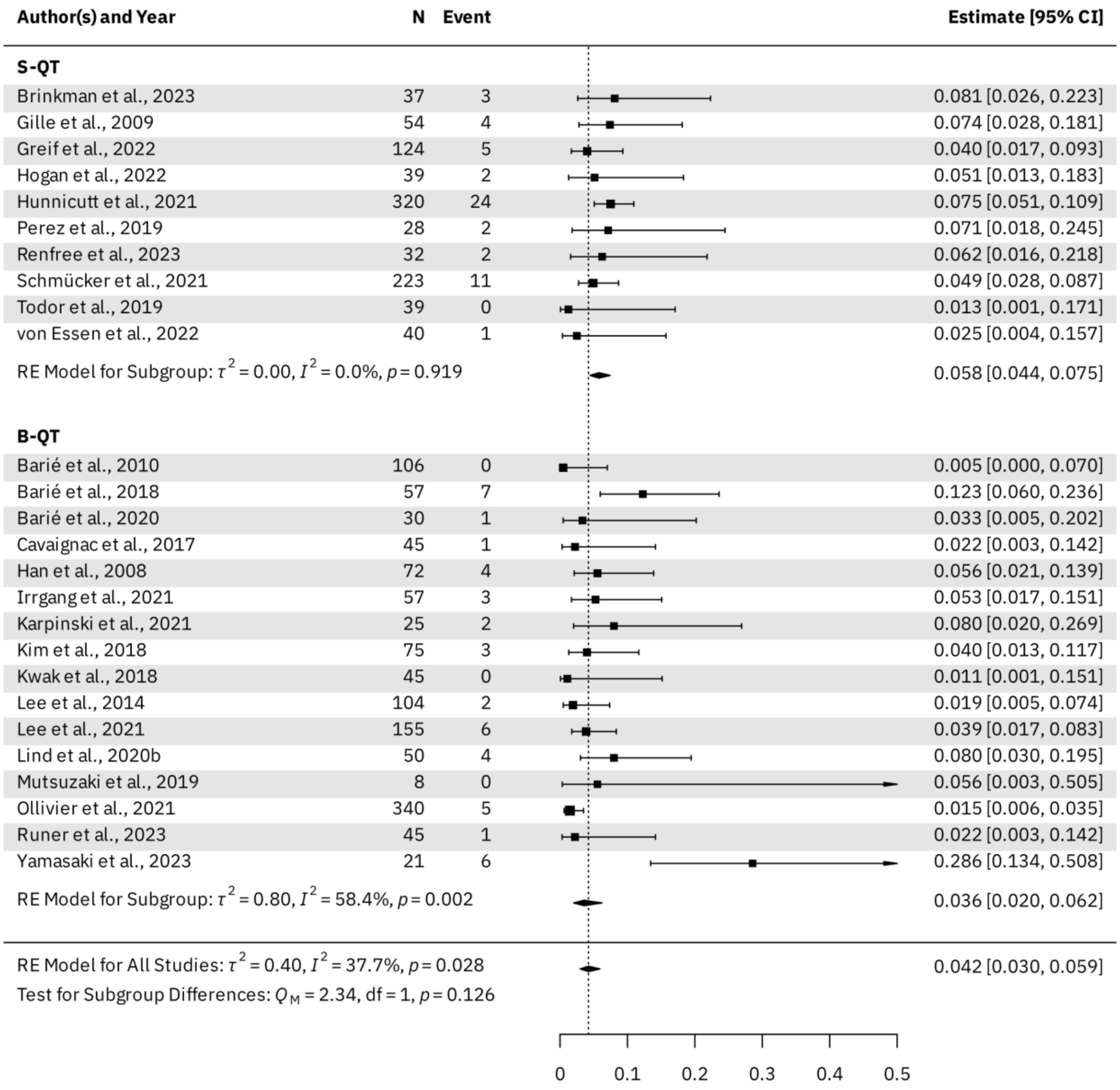

Supplementary File

Supplementary Figure 16 Forest plot of patellar fracture for the B-QT and S-QT subgroups

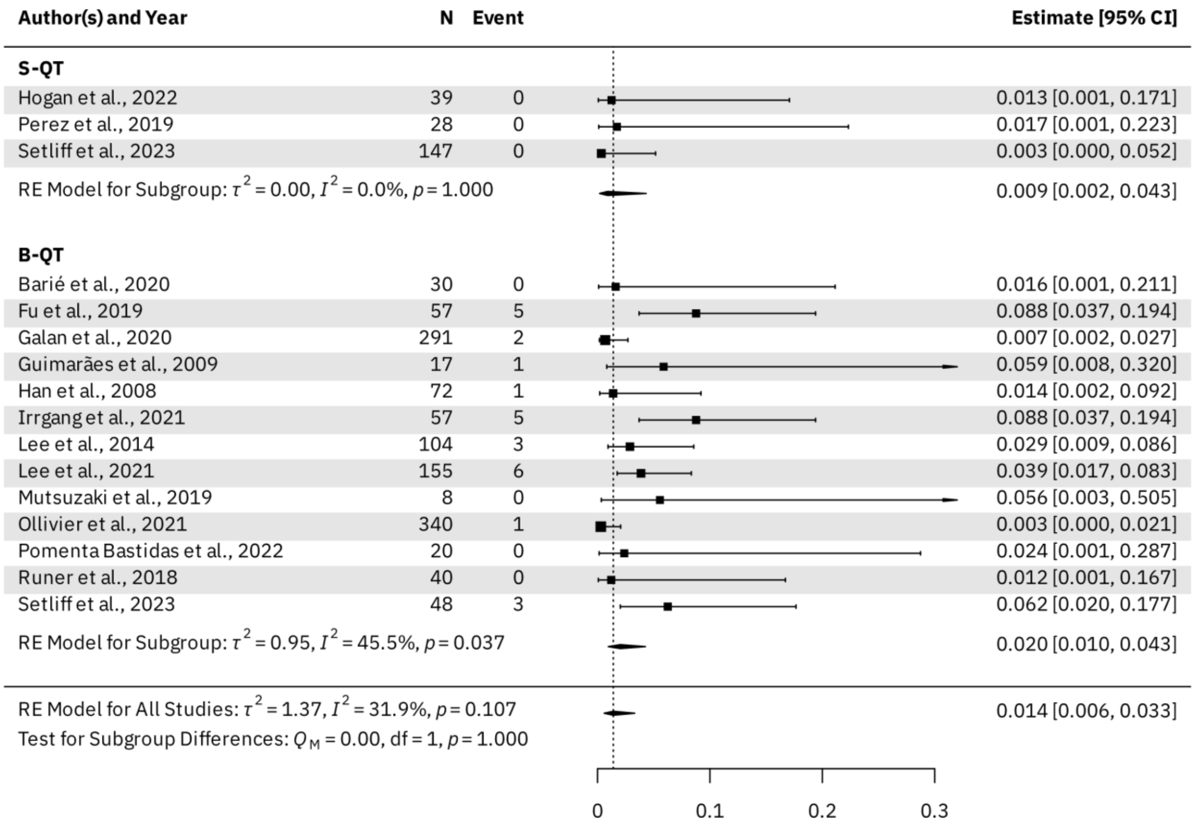

Supplementary File

Supplementary Figure 17 Forest plot of donor site morbidity for the B-QT and S-QT subgroups

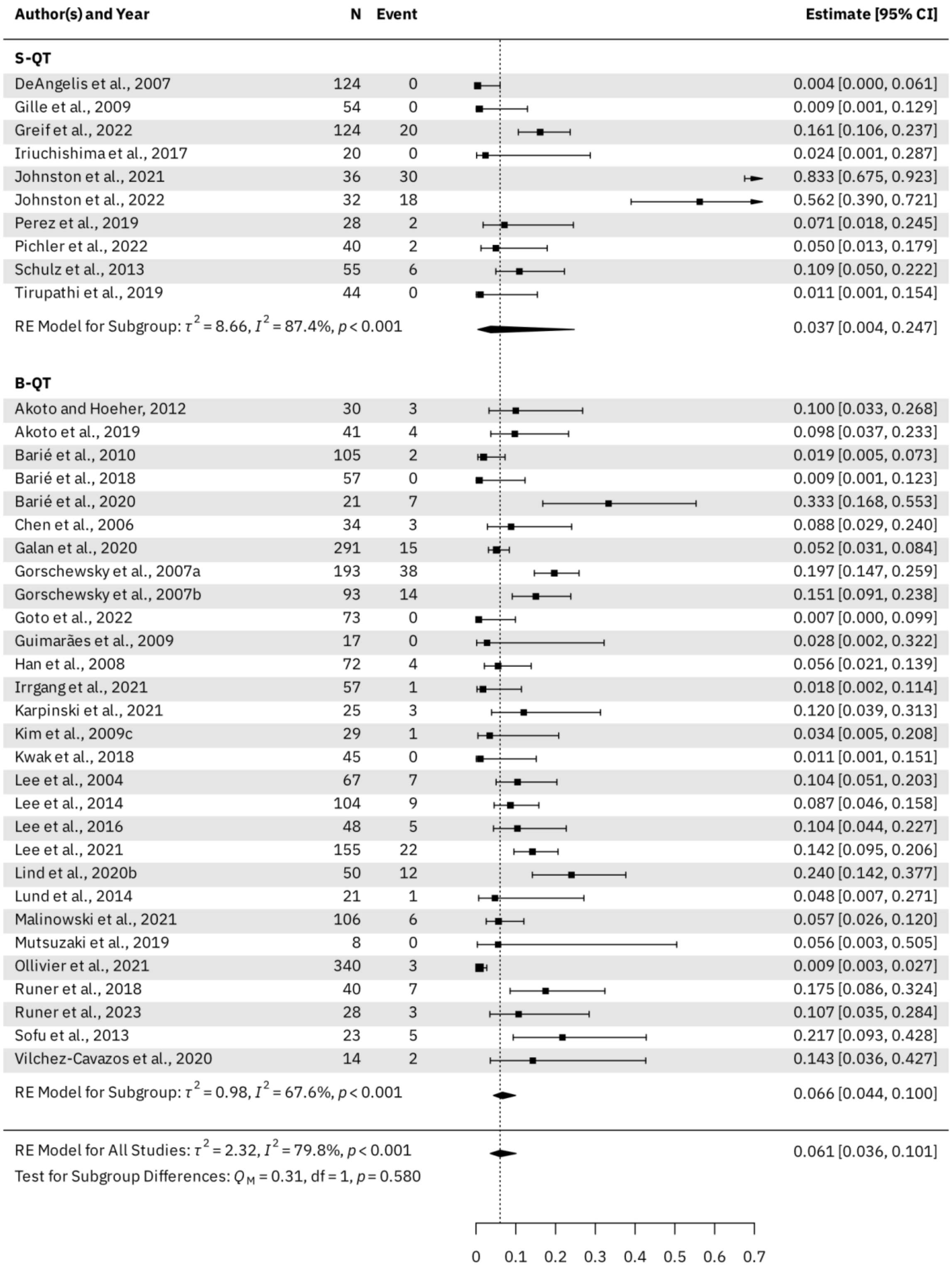

Supplementary File

Supplementary Figure 18 Forest plot of visual analogue scale (VAS) for pain for the B-QT and S-QT subgroups

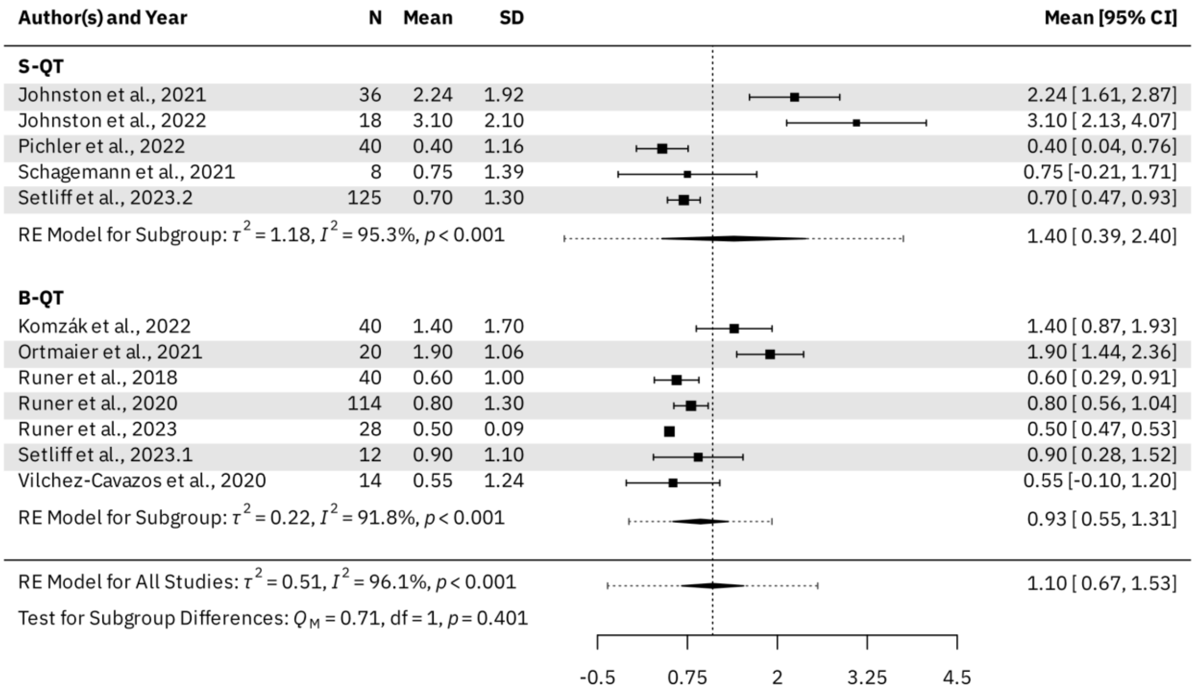

Supplement: Supplementary file 1 [file supplementary_materials.pdf]
